# Spatiotemporal Dynamics in Prespeech Semantic Category Decoding: An Intracranial EEG Study

**DOI:** 10.1523/ENEURO.0254-25.2026

**Published:** 2026-04-21

**Authors:** Ye Jin Park, Jii Kwon, Gyuwon Lee, Kevin Meng, Chun Kee Chung

**Affiliations:** ^1^Department of Interdisciplinary Program in Neuroscience, Seoul National University, Seoul 08826, Republic of Korea; ^2^Department of Brain and Cognitive Sciences, Seoul National University, Seoul 08826, Republic of Korea; ^3^Department of Biomedical Engineering, The University of Melbourne, Victoria 3010, Australia; ^4^Neuroscience Research Institute, Seoul National University Medical Research Center, Seoul 03080, Republic of Korea

**Keywords:** cortical dynamics, intracranial EEG, pre-speech processing, semantic decoding, speech production

## Abstract

Despite advances in brain–computer interfaces, decoding high-level language representations prior to speech remains challenging. While prior efforts have focused on acoustic or articulatory features, how semantic categories are decoded in time and space remains unclear. Here, we investigated how semantic representations unfold over time by analyzing high-gamma (HG; 70–170 Hz) electrocorticography signals from 20 subjects (7 females and 13 males) performing a word-reading task with body- and nonbody-related words. HG activity was examined from word presentation to 500 ms. Group-level time–resolved decoding within each Brodmann area (BA) revealed significant classification accuracy above chance in both hemispheres (*p* < 0.05, FDR-corrected). In the left hemisphere, peak BAs followed a frontal–temporal–occipital–parietal cascade: dorsolateral prefrontal cortex (dlPFC; 50 ms), inferior temporal and fusiform gyri (350–400 ms), and supramarginal gyrus (SMG; 500 ms). In contrast, the right hemisphere exhibited an occipital–temporal–frontal–temporal–parietal sequence: visual and temporal pole (TP) regions (50–100 ms), dlPFC (200 ms), fusiform gyrus (400 ms), and angular gyrus (450 ms). This contrasts with the frontal-initiated cascade of the left hemisphere, underscoring hemispheric differences in the timing of peak decoding loci. Cross-temporal regression revealed predictive interregional engagement. In the left hemisphere, early dlPFC activity (0–150 ms) predicted later SMG responses (300–350 ms). In the right, a strong predictive link emerged from the TP to the angular gyrus (200–300 ms; peak *R*^2^ ≈ 0.70). These findings demonstrate that semantic category decoding relies on temporally structured interregional interactions, revealing distinct hemispheric patterns.

## Significance Statement

This study investigates spatiotemporal dynamics in decoding semantic categories during the prespeech interval using high-resolution intracranial EEG. We reveal a left-hemisphere cascade beginning in frontal areas and extending to temporal, occipital, and parietal regions and a distinct right-hemisphere cascade involving early occipital and temporal pole activity. Cross-temporal regression reveals sustained left-lateral predictive temporal pattern and a brief but high-precision right–hemisphere link. These findings advance our understanding of how semantic categories are constructed in the brain over time and may inform future efforts to develop neural decoding frameworks that operate before speech output.

## Introduction

Speech is an essential tool for communication, thereby survival, enabling individuals to convey their intentions. For individuals with congenital or acquired speech impairments, brain–computer interfaces (BCIs) offer a promising means to restore communication. Recent BCI research has predominantly focused on acoustic and articulatory decoding. Acoustic decoding involves the perception and processing of sound waves (Brumberg et al., 2011; Mugler et al., 2014; Akbari et al., 2019), while articulatory decoding targets the movements of the tongue or the laryngeal motor area during sound production (Dichter et al., 2018; Moses et al., 2019). However, these approaches face limitations when applied to individuals with aphasia, who may lack the ability to articulate speech, underscoring the need to investigate neural mechanisms at the semantic level.

Language processing extends beyond articulation to include complex cognitive processes such as semantic comprehension and conceptual processing (Hickok and Poeppel, 2007; Binder et al., 2009). Semantic categories, which reflect meaning-based groupings of words, play a crucial role in formulating coherent speech. Understanding the neural basis of semantic category processing, particularly during the preparatory stages before speech onset, is therefore essential not only for theoretical models of language but also for informing more inclusive future applications.

Recent intracranial studies have begun exploring the spatiotemporal properties of language. For instance, Zhao et al. (2024) employed stereotactic EEG to examine sublexical encoding in Chinese character reading, identifying cortical dynamics linked to orthographic-to-phonological mapping. While this work identified key dynamics, its focus on character-specific sublexical components limits its generalizability (Zhao et al., 2024). In contrast, the current study focuses on broad, conceptual-level semantic categories, which are universally applicable across languages. Unlike studies focused on static activation or structural connectivity, we analyze predictive interregional dynamics during the prespeech period using high-resolution electrocorticography (ECoG).

While functional magnetic resonance imaging (fMRI) studies highlight the broad spatial network involved in language processing, event-related potential findings underscore the highly time-sensitive nature of these processes, together suggesting that language arises from rapid, distributed neural interactions. Previous fMRI studies linked semantic processing to the posterior superior temporal gyrus (STG), posterior middle temporal gyrus (MTG), angular gyrus, and inferior frontal gyrus (Friederici, 2011; Huth et al., 2016; Xu et al., 2017). Temporal studies show that high-level language processing occurs quickly after word presentation and before speech. Semantic processing, marked by the N400 (∼250 ms), reflects how well a word fits its context—larger N400 amplitudes indicate greater integration difficulty. This disconnect underscores the need for research that unifies spatial and temporal aspects of language processing.

Despite these advances, the specific brain areas and their temporal interactions that support decodable semantic category features prior to speech remain unclear. Different brain regions process information both serially and in parallel over time, but the specific brain areas involved at different time points remain unclear. To address this gap, our research utilized ECoG, which offers high spatiotemporal resolution. The high-gamma (HG) band is particularly advantageous due to its higher spatial and temporal resolution compared with lower-frequency bands and its strong correlation with cognitive tasks, including motor and speech activities (Crone et al., 2006).

In this study, we investigate the spatiotemporal dynamics of semantic category processing during the prespeech window using HG ECoG signals. Subjects engaged in a word-reading task involving “body-part” and “nonbody-part” categories. While the task required subjects to overtly read individual words, we posited that semantic categorization would occur prior to articulation, consistent with prior findings on early-stage language comprehension (Brown and Hagoort, 1993; Friederici, 2011; Brouwer et al., 2017). By analyzing neural activity from stimulus onset to 500 ms—prior to speech initiation—we aim to identify the temporal cascade and interregional interactions underpinning semantic processing.

## Materials and Methods

### Subjects

Twenty subjects (7 females; average age 34) with medically intractable epilepsy who had been temporarily implanted with intracranial electrodes for monitoring purposes participated in this study after providing written informed consent. All recordings were performed during the clinical monitoring period, and study participation did not interfere with the subjects' diagnostic or therapeutic procedures. Data from one of these subjects were excluded from all analyses because the individual's speech-onset time was an outlier (>2 SD). The study protocol was approved by the Institutional Review Board of Seoul National University Hospital (H-2011-087-1173). All subjects were native Korean speakers with no history of language deficits. According to Annett's hand preference questionnaire, which was conducted during a neuropsychological evaluation prior to surgery, 1 was left-handed, 2 were ambidextrous, and the remaining 17 were right-handed. Subject details are provided in Table 1. There was no significant difference in the number of electrodes between the left and right hemispheres (left, 686; right, 630).

**Table 1.** Demographics, clinical characteristics, and number of electrodes

Subject	Demographics		Clinical characteristics			
	Age	Gender	Handedness	Hemisphere	Epilepsy type	Number of electrodes
1	25–30	M	Right	Left	TLE	58
2	20–25	M	Right	Left	FTLE	68
3	45–50	M	Left	Left	TLE	68
4	20–25	F	Right	Left	TLE	72
5	20–25	F	Right	Right	FTLE	60
6	45–50	M	Right	Left	FTLE	76
7	15–20	F	Right	Left	TLE	92
8	30–35	M	Right	Left	FLE	40
9	20–25	M	Right	Right	FLE	44
10	50–55	M	Right	Left	TLE	76
11	55–60	M	Right	Right	FTLE	38
12	35–40	F	Right	Right	OLE	42
13	35–40	M	Right	Right	TLE	84
14	35–40	M	Right	Right	Tumor	52
15	45–50	F	Right	Left	TLE	60
16	20–25	F	Right	Right	TLE	106
17	25–30	F	Right	Right	TLE	68
18	30–35	M	Ambidextrous	Right	TLE	68
19	30–35	M	Ambidextrous	Left	TLE	76
20	25–30	M	Right	Right	TLE	68
							

TLE, temporal lobe epilepsy; FTLE, frontal and temporal lobe epilepsy; OLE, occipital lobe epilepsy.

### Electrode localization and data recording

**Figure 1.**
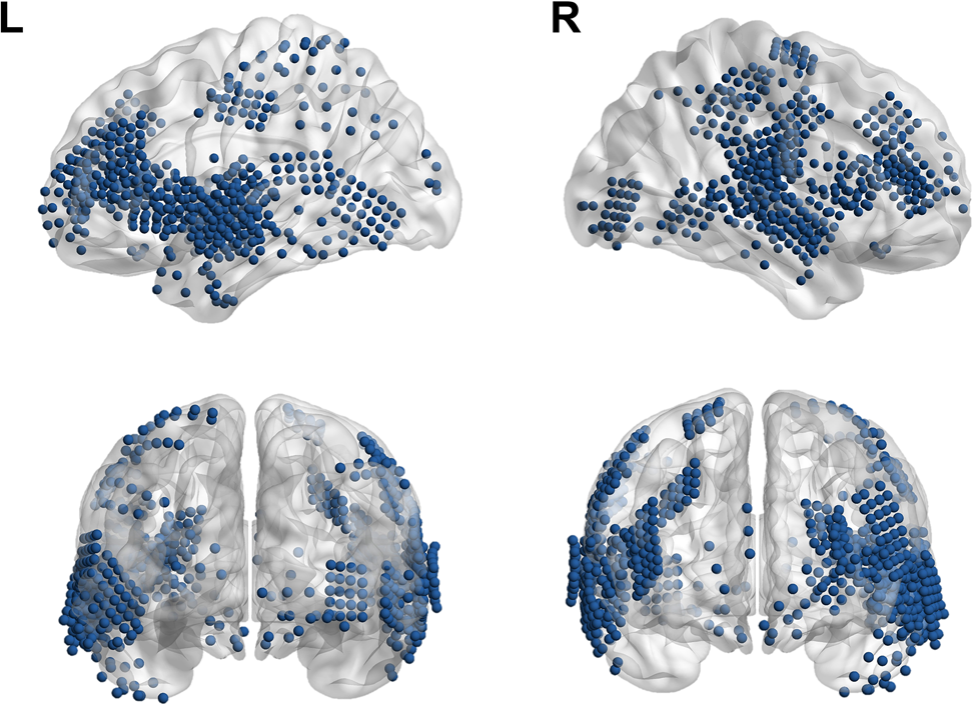
Lateral sagittal (top) and coronal (bottom, left; posterior, right; anterior) views of grid and strip electrodes from twenty subjects, rendered on normalized cortical surfaces. Electrodes were grouped in brain areas based on the Talairach coordinates, as illustrated in Table 2.

High-density electrodes with stainless steel contacts (2 mm diameter, spaced 5 mm apart; PMT) were used solely for clinical purposes and placed on the cortical surface. Prior to the electrode placement, MRI were conducted using either a Magnetom Trio, Verio 3 Tesla (Siemens), or a Signa 1.5 Tesla scanner (GE HealthCare), along with computed tomography (CT) using a Somatom sensation device (64 eco; Siemens). After the electrodes were inserted, further CT and MRI scans were performed to verify their positions. The alignment of preoperative MRI and postoperative CT images to determine electrode locations was carried out using the CURRY software (version 7.0; Compumedics Neuroscan). Intracranial electrical activity was recorded at a 2 kHz sampling rate with a Neuvo amplifier, managed by either the ProFusion EEG software (Compumedics) or CURRY software. As shown in Figure 1, BrainNet Viewer was used to visualize electrode location (Xia et al., 2013). Electrodes were grouped into brain areas according to Talairach coordinates (Lacadie et al., 2008), and ROIs were chosen based on findings from previous speech studies (Hickok and Poeppel, 2007; Friederici, 2011; Huth et al., 2016; Hagoort, 2017; Xu et al., 2017; Nagata et al., 2022), as illustrated in Table 2.

**Table 2.** Electrodes grouped by brain areas based on Talairach coordinates, excluding depth electrodes and bad channels with high impedance

Brain region	Left (L)/right (R)	BA	Number of electrodes
Anterior prefrontal cortex	L	10	91
Anterior prefrontal cortex	R	10	27
dlPFC	L	46, 9	53
dlPFC	R	46, 9	43
Pars opercularis	L	44	17
Pars opercularis	R	44	20
Pars triangularis	L	45	47
Pars triangularis	R	45	16
Pars orbitalis	L	47	28
Pars orbitalis	R	47	9
Premotor/supplementary motor cortex	L	6	21
Premotor/supplementary motor cortex	R	6	39
Primary motor cortex	L	4	14
Primary motor cortex	R	4	20
Primary somatosensory cortex	L	1, 2, 3	18
Primary somatosensory cortex	R	1, 2, 3	45
SMG	L	40	11
SMG	R	40	52
Angular gyrus	L	39	14
Angular gyrus	R	39	7
STG	L	22	54
STG	R	22	56
MTG	L	21	88
MTG	R	21	49
FG	L	37	23
FG	R	37	19
Others	L	N/A	91
Others	R	N/A	90

The details are summarized by brain region, hemisphere, BA, and number of electrodes.

### Experimental design

**Figure 2.**
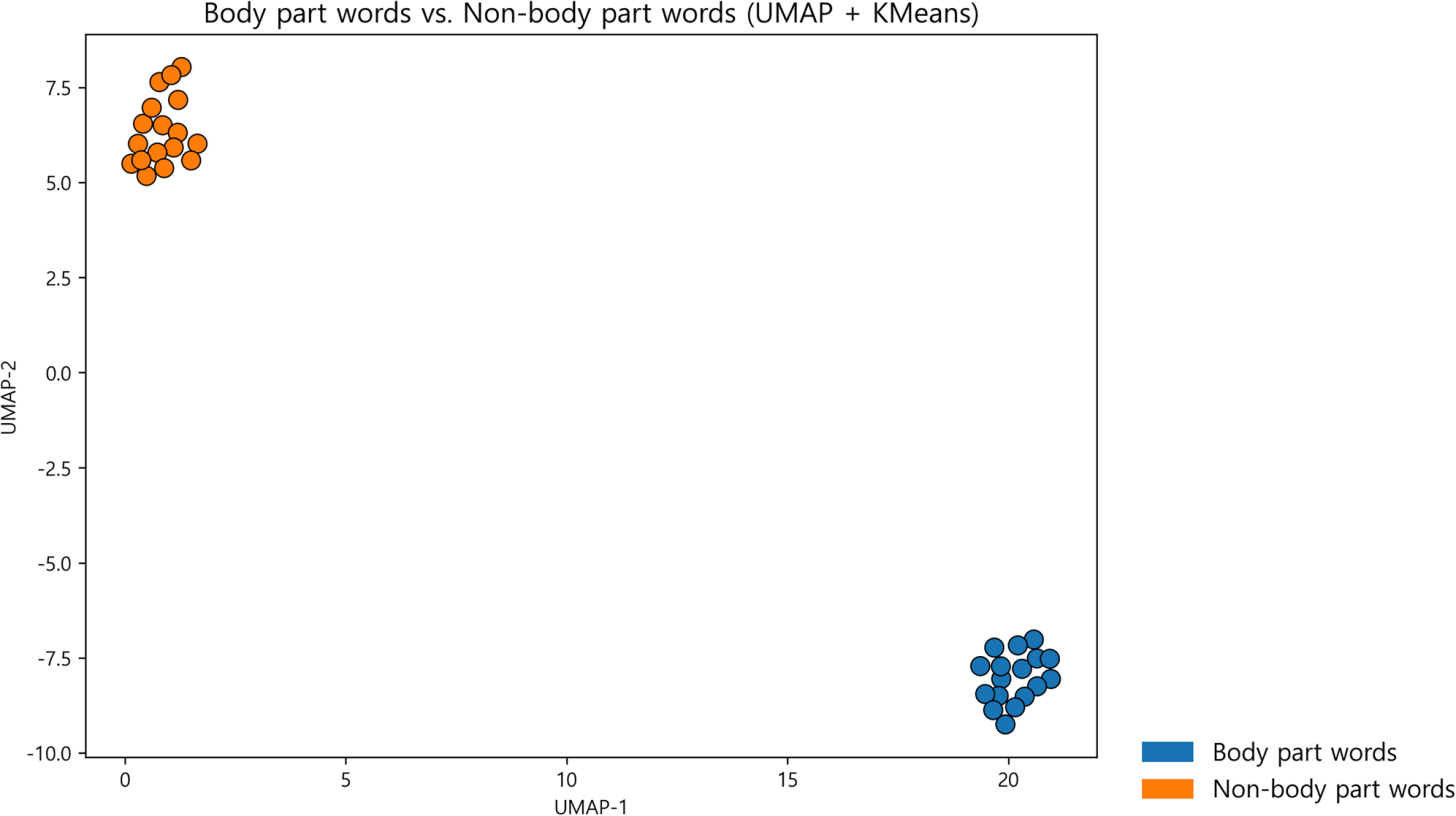
Word embeddings (sentence BERT-based model trained on Korean) were projected to two dimensions using UMAP and partitioned with *K*-means (*k* = 2) for visualization. Colors indicate cluster assignment; word–cluster mappings are provided in Extended Data Figure 2-1. Additional analyses of psycholinguistic properties are provided in Extended Data Figures 2-2 and 2-3.

Subjects were instructed to read aloud a Korean word, which was grouped semantically—as body parts or nonbody parts (Extended Data Fig. 2-1). To visualize the relationship between these two categories, all stimulus words were embedded using a Korean Sentence-BERT-based model and projected into a two-dimensional space with UMAP followed by *K*-means clustering (*k* = 2; Fig. 2). The resulting clusters corresponded closely to the predefined categories, confirming that the two groups occupied distinct regions in semantic space.

10.1523/ENEURO.0254-25.2026.f2-1 Figure 2-1 A categorized list of 34 words (body-part vs. non-body-part) was used in the speech-production task; the words were randomly displayed on the screen one at a time. Download Figure 2-1, DOCX file.

10.1523/ENEURO.0254-25.2026.f2-2 Figure 2-2 Control analyses of low-level structural properties across stimulus words. Phoneme-level distance and syllable count differences were examined to assess potential low-level confounds. Phoneme-level distance was computed by decomposing each word into Korean Hangul phoneme (jamo) sequences and calculating the Levenshtein distance between sequence. Orthographic distance was computed as the Levenshtein edit distance between Hangul syllable characters, and syllable difference was defined as the absolute difference in syllable counts (|Δ|) between word pairs. (A) Pairwise comparisons between within- and between-category word pairs show no systematic increase in phoneme-level distance, orthographic distance, or syllable differences across semantic categories. (B) Representational dissimilarity matrices (RDMs) constructed from semantic category labels (0 = within-category, 1 = between-category), phoneme-level distance, orthographic distance, and syllable count differences reveal a clear categorical block structure only in the semantic RDM, whereas the phoneme-level, orthographic, and syllable RDMs do not exhibit corresponding organization. (C) Spearman correlations computed across all unique pairwise dissimilarity values indicate minimal association between semantic category structure and phoneme-level distance (ρ = 0.069), orthographic distance (ρ = 0.042) or syllable difference (ρ = 0.038), while phoneme-level distance and syllable difference show a moderate correlation (ρ = 0.553). These results indicate that the semantic categorical structure is not accounted for by the measured low-level phonological or syllabic properties. Download Figure 2-2, TIF file.

**Figure 3.**
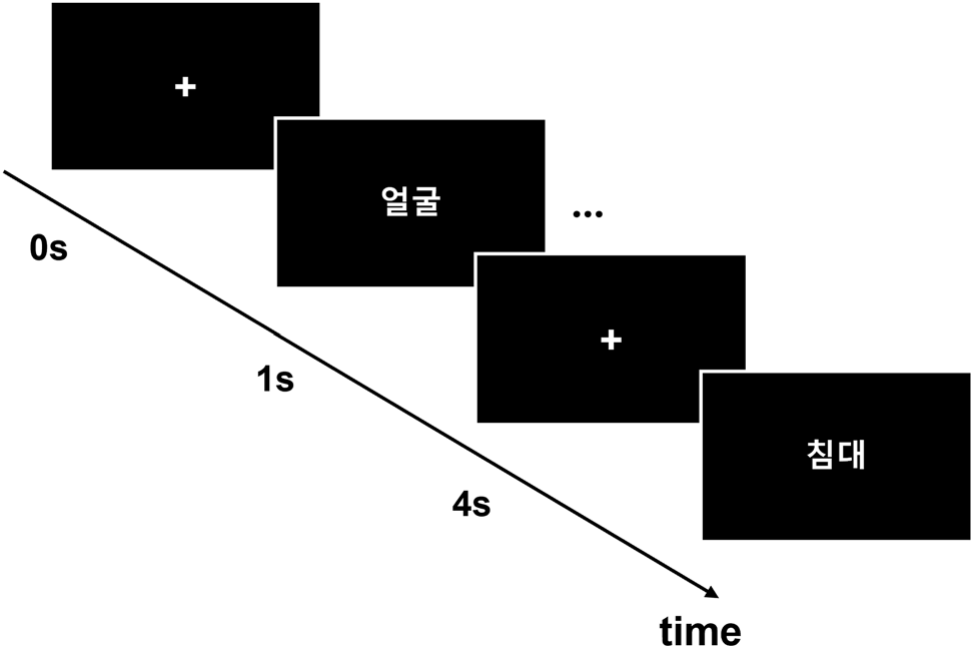
The experiment design for the Korean word-reading task included a fixation cross displayed for 1 s, followed by a word displayed for 3 s (word list in Extended Data Fig. 2-1). Speech-onset distributions for each subject are shown in Extended Data Figure 3-1.

10.1523/ENEURO.0254-25.2026.f2-3 Figure 2-3 Psycholinguistic comparison of stimulus sets. Scatter plots show word frequency (Zipf scale, Korean wordfreq norms; left) and concreteness ratings (English norms via translation; right) for body-part and non-body words. Word frequency did not differ significantly between categories (Welch’s p = 0.10; Mann–Whitney p = 0.15), whereas concreteness was significantly higher for body-part words (Welch’s p = 0.001; Mann–Whitney p = 0.023; Cohen’s d = 1.32). Error bars indicate mean ± standard error. Download Figure 2-3, TIF file.

Potential psycholinguistic confounds, including phoneme-level distance, syllable count (i.e., word length) differences, orthographic distance, word frequency, and concreteness, were quantified and compared across categories (Extended Data Figs. 2-2, 2-3). These words were selected based on the vocabulary typical of children aged 6–9 years (Johnson et al., 2016) and from terms used in assistive communication devices for intensive care unit patients on mechanical ventilation (Duffy et al., 2018). The focus on body-part versus nonbody-part words follows prior semantic decoding studies that employed categorical distinctions such as body-related concepts (Simanova et al., 2010; Mazurchuk et al., 2024) and was chosen both for theoretical relevance and for practical applicability to future communication aids for patients with speech impairments.

Each subject completed a single presentation of each word, yielding 34 trials in total per subject. Visual cues included a fixation cross for 1 s and a word displayed for 3 s (Fig. 3). These cues and intracranial recordings were synchronized using StimTracker (Cedrus). Audio was simultaneously recorded at 16 kHz to determine the speech onset.

10.1523/ENEURO.0254-25.2026.f3-1 Figure 3-1 Kernel density estimates of speech-onset times for each subject. Time is referenced to fixation onset (0–3 s), with the dashed vertical line at 1 s marking word presentation. Density peaks across subjects typically fall between 1.5 and 2.5 s, corresponding to 0.5–1.5 s post-word onset. Subject 11 was excluded from further analyses due to a speech-onset distribution exceeding 2 SD from the group mean. Download Figure 3-1, TIF file.

### Preprocessing

MATLAB (R2022b, MathWorks) and Python (version 3.9) were used for the analysis. The intracranial data were referenced to the common average and were notch filtered at 60, 120, and 180 Hz to remove power noise and its harmonics. Electrodes showing high impedance (>10 kΩ), as indicated by the impedance check during clinical recording, were excluded from further analysis. Trials containing interictal epileptiform discharges, defined as transient spikes or sharp waves with durations of 20–200 ms and amplitudes exceeding the background by more than twofold (Flach et al., 2024), were also discarded. Time–frequency analysis showing spectral dynamics between the two categories during the word-reading task, performed using continuous wavelet transform analysis, is illustrated in Extended Data Figure 4-1. Subsequently, the data underwent bandpass filtering into the HG band (70–170 Hz). HG amplitudes, normalized to a prestimulus baseline, were then used for feature selection.

### Feature selection and decoding

**Figure 4.**
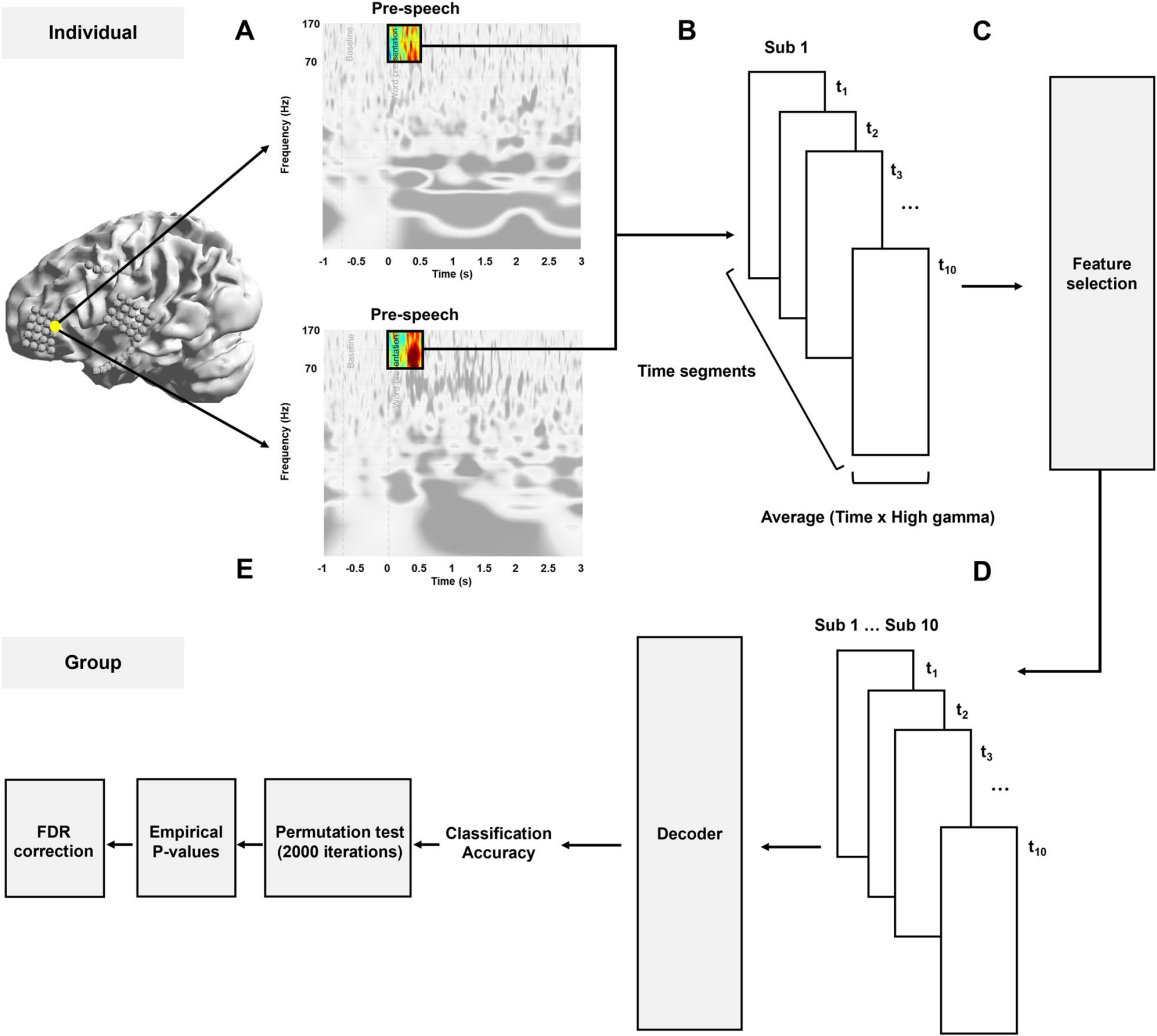
Outline of the process from preprocessed data to group-level decoding. ***A***, For each electrode, time–frequency analysis of the prespeech interval (illustrated in Extended Data Fig. 4-1) was performed, and HG (70–170 Hz) power was averaged to yield a single amplitude trace (color inset). ***B***, The HG trace was segmented into 10 overlapping windows of 100 ms, advanced in 50 ms steps (*t*_1_–*t*_10_), covering 0–500 ms from word onset. ***C***, Feature selection: 70% of the trials were reserved for evaluating each channel independently with fivefold stratified linear SVM cross-validation. Channels whose mean accuracy exceeded chance (>0.50) were retained. ***D***, Retained channels were concatenated within each BA and entered into the multichannel decoder. Classification accuracy, averaged over five cross-validation folds, produced the time-resolved curves and cortical maps shown in Figures 5 and 6. ***E***, Statistical validation of decoding accuracy. Observed accuracies for each BA and time window were evaluated against a 2,000-iteration label–shuffled permutation distribution to obtain empirical *p* values. Benjamini–Hochberg FDR (*α* = 0.05) correction was then applied across time windows within each BA to control for multiple comparisons.

To assess the temporal contribution of neural features, time–frequency transformed ECoG signals were analyzed with 100 ms sliding windows (Schönmann et al., 2025; Zhang et al., 2025) advanced in 50 ms steps from stimulus onset to 500 ms (Fig. 4). To avoid information leakage, 70% of the trials were allocated to feature selection. Each channel was then evaluated independently using a linear support vector machine (SVM) with fivefold stratified cross-validation, and channels whose mean decoding accuracy exceeded chance (>0.50) were retained for subsequent multichannel analyses (Proix et al., 2022).

10.1523/ENEURO.0254-25.2026.f4-1 Figure 4-1 Time-frequency analysis showing spectral dynamics between the two semantic categories (body part vs. non-body part) during the word reading task, performed using continuous wavelet transform analysis. The period from -1 s to 0 s represents fixation, -0.3 s to 0 s serves as the baseline, and 0 s to 3 s represents the word presentation. The black square indicates the pre-speech period. This example is from an individual subject with a left hemisphere implant. Download Figure 4-1, TIF file.

### Time-resolved multichannel decoding and classifier comparison

Group-level decoding was conducted by aggregating the selected channels within each Brodmann area (BA) across subjects. For every window the concatenated features entered three classifiers—linear SVM, linear discriminant analysis, and random forest. The linear SVM achieved the highest mean accuracy and was adopted for the main analyses, producing time-resolved accuracy curves for each BA and, after grouping, for each cortical lobe (Extended Data Fig. 5-1). To assess statistical significance, decoding accuracies (five cross-validation folds) were tested against a 2,000-iteration label–shuffled permutation distribution. For each BA and time window, empirical *p* values were computed relative to the corresponding null distribution. Benjamini–Hochberg false discovery rate (FDR; *α* = 0.05) was applied across time windows within each BA to control for multiple comparisons. BAs that remained significant are visualized as color-coded patches on inflated cortical surfaces.

### Cross-temporal regression analysis

To further assess the temporal relationships between brain regions, we conducted cross-temporal linear regression analyses in two ways: (1) with all trials pooled (combined version) and (2) separately for body-part and nonbody trials (category-specific version). BAs whose decoding peaks were significant above, Time-resolved multichannel decoding and classifier comparison, were labeled early (≤250 ms) or late (>250 ms), based on neurophysiological and theoretical considerations. The 250 ms threshold aligns with the onset of the N400 component—widely regarded as a neural signature of semantic integration—which typically emerges around this time (Friederici, 2011; Kutas and Federmeier, 2011). This cutoff is also consistent with temporally structured models of language processing that distinguish early lexico-semantic access from later semantic unification processes (Hagoort, 2017). For each pair, we computed the coefficient of determination (*R*^2^) for every source-time × target-time combination. Statistical significance of each *R*^2^ value was estimated from 1,000 permutation-based null distributions, and the resulting *p* values were adjusted with the Benjamini–Hochberg FDR correction (α = 0.05). The resulting combined and category-specific *R*^2^ matrices reveal when activity in an early BA predicts later activity in a distant lobe, mapping the temporal flow of semantic information. For visualization purposes, we also computed the mean HG amplitude across all subjects for selected source–target BA pairs, enabling comparison of raw signal dynamics with regression and decoding results

### Code accessibility

The code described in the paper is freely available online at https://github.com/jinnilog/semantic-category-2025.git. The code is available as Extended Data.

10.1523/ENEURO.0254-25.2026.d1 Data 1 Code used in the analyses described in the manuscript. The file *2.4_preprocessing_sub16.m* provides an example of the preprocessing procedures applied to the ECoG data. The script *2.5-2.7_decoding_semantic_left.py* was used for feature selection, time-resolved decoding, cross-temporal regression analysis, and figure generation. Download Data 1, ZIP file.

## Result

### Behavioral performance

The average speech-onset time across all subjects was 881 ms (±228 ms), and the earliest onset observed was 500 ms. Accordingly, the 0–500 ms interval was taken as the prespeech window. The distribution of individual onset times is displayed in Extended Data Figure 3-1.

### Time-resolved semantic category decoding performance

**Figure 5.**
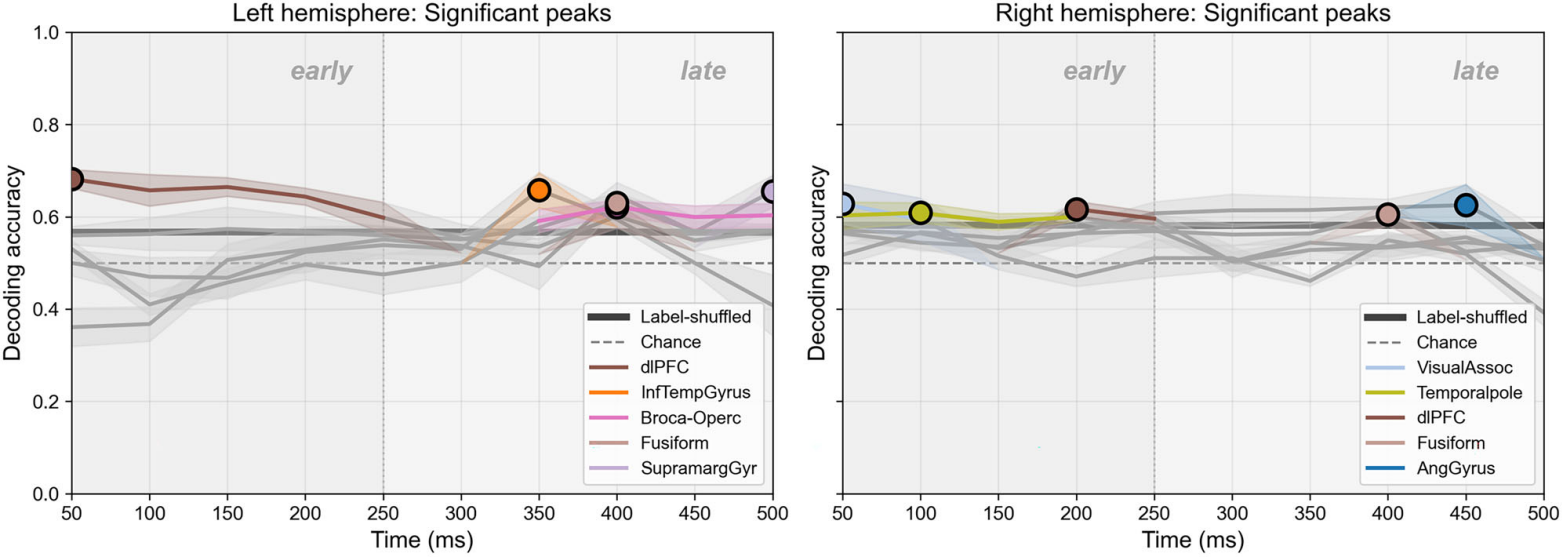
Time-resolved decoding of semantic category in the left and right hemispheres. HG activity (70–170 Hz) was used to decode body-part versus nonbody words using 100 ms windows advanced in 50 ms steps from 0 to 500 ms before speech onset. Lines indicate mean decoding accuracy for each BA, with shaded bands representing ±standard error. The gray dashed horizontal line marks chance level (0.5), and the *y*-axis ranges from 0 to 1. Decoding accuracies (five cross-validation folds) were tested against a 2,000-iteration label–shuffled permutation distribution; empirical *p* values were computed for each BA and time window, and Benjamini–Hochberg FDR correction (α = 0.05) was applied across time windows within each BA. Circles denote peak time points that survived FDR correction. In addition, a time-shuffled permutation analysis was performed, and only time intervals exceeding the corresponding time-shuffle threshold are shown in color. The vertical divider separates early (≤250 ms) and late (>250 ms) prespeech intervals. Extended Data Figure 5-1 shows the corresponding classifier comparison, and Extended Data Figure 5-2 presents decoding minima evaluated with identical statistical criteria, illustrating the asymmetric distribution of significant decoding above and below chance. Extended Data Figure 5-3 presents a control analysis excluding number words from the nonbody-part category, which yielded a comparable spatiotemporal decoding pattern with differences likely reflecting reduced trial counts. Extended Data Figure 5-4 demonstrates replication of left-hemisphere decoding after inclusion of an additional subject, showing a consistent temporal and regional pattern under the same analytical procedures.

**Figure 6.**
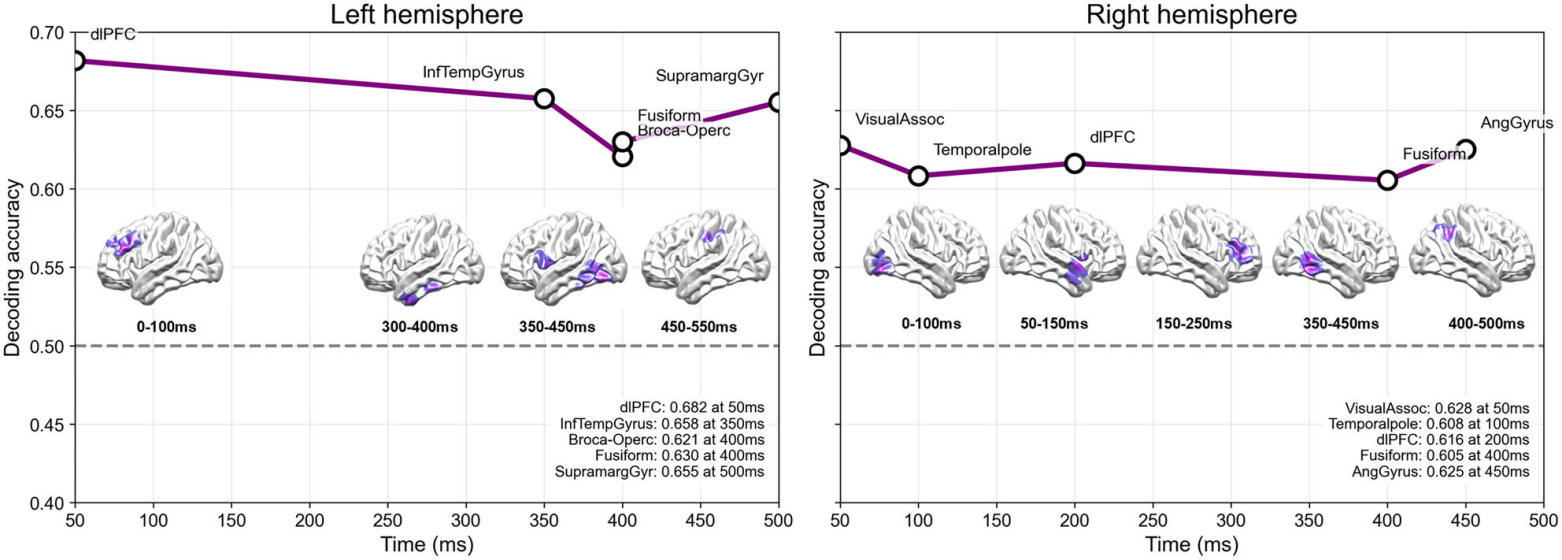
Temporal progression of significant decoding peaks across regions. Statistically validated decoding maxima for each BA in the left and right hemispheres are shown. Only time points that survived both the label-shuffled permutation test (FDR-corrected) and the time-shuffle threshold are included. Brain renderings below show the anatomical locations of BAs exhibiting significant decoding. Circles mark the significant time points for each BA, and connecting lines illustrate the relative temporal ordering across regions. The dashed horizontal line marks chance level (0.5), and the *y*-axis shows decoding accuracy.

Semantic category decoding of HG activity (70–170 Hz) during the 0–500 ms prespeech window produced the time-resolved accuracy curves and peak summaries shown in Figures 5 and 6. Figure 5 presents the full time-resolved decoding profiles for each BA, whereas Figure 6 summarizes the statistically validated peak time points and their temporal progression across regions. Considering the highest-performing area within each lobe, the left hemisphere showed a clear temporal cascade: dorsolateral prefrontal cortex (dlPFC) at 50 ms (accuracy = 0.682; *p* = 0.0007); inferior temporal gyrus (ITG) at 350 ms (0.658, *p* = 0.0336); pars opercularis and fusiform gyrus (FG) at 400 ms (0.621, *p* = 0.0057; 0.630, *p* = 0.0310); and supramarginal gyrus (SMG) at 500 ms (0.655, *p* = 0.0046), delineating a frontal–temporal–occipital–parietal progression of maximal decoding performance.

10.1523/ENEURO.0254-25.2026.f5-1 Figure 5-1 Box-plot comparison of decoding accuracy (averaged over 0–500 ms post-stimulus) across classifiers—Support Vector Machine (SVM), Linear Discriminant Analysis (LDA), and Random Forest (RF)—for the best-performing BA within each lobe. Left and right panels show results from the left and right hemispheres, respectively. Overall, decoding performance was comparable across classifiers, with no statistically significant differences observed in most lobes. However, in the right hemisphere's Occipital lobe, both SVM and LDA achieved significantly higher accuracy than RF (FDR-corrected p = 0.0136 and 0.0107, respectively). No other classifier pairwise comparisons reached significance following FDR correction (Asterisks indicate significant pairwise differences between classifiers (p < 0.05, FDR-corrected, within-lobe). Download Figure 5-1, TIF file.

10.1523/ENEURO.0254-25.2026.f5-2 Figure 5-2 (A) Significant decoding troughs below chance level. Statistically significant decoding minima are shown for each Brodmann area (BA) in the left and right hemispheres. Minima were evaluated against the lower tail of the 2000-iteration label-shuffled permutation distribution with FDR correction across time windows within each BA, and only time points that also passed the time-shuffle threshold are displayed. Circles indicate significant troughs. (B) Asymmetric temporal distribution of significant decoding above and below chance. Binary matrices display time windows showing statistically significant decoding above (top panels) and below (bottom panels) chance for each BA in the left and right hemispheres. Identical statistical criteria were applied in both directions (2000-iteration label-shuffled permutation with FDR correction across time windows within each BA, combined with the time-shuffle threshold). Yellow cells indicate time points meeting both criteria; purple cells indicate non-significant windows. Download Figure 5-2, TIF file.

10.1523/ENEURO.0254-25.2026.f5-3 Figure 5-3 Decoding accuracy after excluding number words. Time-resolved decoding results for the left and right hemispheres after removing numerals from the non-body-part category. The number of body-related trials was randomly resampled (500 iterations) to match the reduced control set. Colored lines show decoding accuracy (± SEM) for significant Brodmann areas; the dark gray line indicates the mean shuffled control (95th percentile ± SEM). The overall spatiotemporal pattern remained consistent with the full analysis, showing early bilateral dorsolateral prefrontal and later left fusiform and pars opercularis activity. Because excluding number words reduced the number of available trials by nearly half, fewer Brodmann areas reached statistical significance, reflecting lower statistical power rather than a change in the underlying decoding pattern. Download Figure 5-3, TIF file.

10.1523/ENEURO.0254-25.2026.f5-4 Figure 5-4 Replication of left-hemisphere decoding peaks after inclusion of an additional subject. Time-resolved decoding results in the left hemisphere are shown before (left panel) and after (right panel) inclusion of an additional subject, using identical preprocessing, decoding, and permutation-based statistical procedures. Circles indicate peak time points that survived the label-shuffled permutation test (FDR-corrected) and time-shuffle threshold. The temporal profile and regional pattern of significant peaks, particularly in dlPFC and fusiform cortex, remain consistent after inclusion of the additional dataset. Download Figure 5-4, TIF file.

In the right hemisphere, the best-performing FDR–significant area in each lobe emerged in a different order: visual association cortex (VAC) at 50 ms (0.628, *p* = 0.0465), temporal pole (TP) at 100 ms (0.608, *p* = 0.0036), dlPFC at 200 ms (0.616, *p* = 0.0023), FG at 400 ms (0.605, *p* = 0.0027), and angular gyrus at 450 ms (0.625, *p* = 0.0056; Table 3). This occipital–temporal–frontal–occipital–parietal sequence contrasts with the frontal-initiated cascade observed in the left hemisphere, underscoring hemispheric differences in the timing of peak decoding loci. To further assess the reliability of the decoding results, significant decoding troughs below chance were evaluated using the identical statistical criteria; these troughs were rare and temporally restricted (Extended Data Fig. 5-2), indicating that decoding was not symmetrically distributed around chance. The overall spatiotemporal pattern was robust across control analyses excluding number words from the nonbody-part category (Extended Data Fig. 5-3), performed to assess whether differences in concreteness could influence decoding. The resulting pattern showed similar early and late decoding peaks despite the smaller number of trials.

**Table 3.** Peak decoding performance across BAs with FDR-corrected significance during the 0–500 ms prespeech window

Brain region	BA	Peak time (ms)	Peak accuracy	*p* value (FDR)
Left-dlPFC(dorsal)	9	50	0.682	0.0007
Left-InfTempGyrus	20	350	0.658	0.0336
Left-SupramargGyr	40	500	0.655	0.0046
Left-Fusiform	37	400	0.63	0.031
Left-pars opercularis	44	400	0.621	0.0058
Left-PrimMotor	4	250	0.604	0.0058
Left-VisualAssoc	19	350	0.586	0.0046
Left-AngGyrus	39	500	0.564	0.0189
Left-Pars triangularis	45	300	0.564	0.0007
Left-Temporalpole	38	350	0.559	0.031
Left-MedTempGyrus	21	150	0.554	0.0007
Left-SupTempGyrus	22	450	0.545	0.0007
Left-ParsOrbitalis	47	350	0.529	0.031
Left-AntPFC	10	150	0.529	0.0154
Right-PrimVisual	17	500	0.681	0.0082
Right-VisualAssoc	19	50	0.628	0.0465
Right-AngGyrus	39	450	0.625	0.0056
Right-dlPFC(lat)	46	200	0.616	0.0023
Right-Temporalpole	38	100	0.608	0.0036
Right-Fusiform	37	400	0.605	0.0027
Right-SupTempGyrus	22	150	0.595	0.0051
Right-PrimAuditory	41	200	0.583	0.0051
Right-MedTempGyrus	21	350	0.577	0.0016
Right-SecVisual	18	200	0.575	0.0027
Right-Pars triangularis	45	50	0.574	0.0051
Right-ParsOrbitalis	47	350	0.572	0.0097
Right-FrontEyeFields	8	500	0.566	0.0136
Right-PrimSensory	1	300	0.565	0.0051
Right-AntPFC	10	450	0.565	0.0006
Right-dlPFC(dorsal)	9	400	0.561	0.0015
Right-PreMot + SuppMot	6	50	0.535	0.0036

The table lists each region's peak time, decoding accuracy, and FDR-adjusted *p* value. Extended Data Table 3-1 summarizes electrode coverage for occipitotemporal regions overlapping with the EBA.

10.1523/ENEURO.0254-25.2026.t3-1 Table 3-1 Electrode coverage in occipitotemporal regions overlapping with the extrastriate body area (EBA), confirmed across subjects included in the decoding analyses. Download Table 3-1, DOCX file.

### Interregional predictive dynamics in prespeech

**Figure 7.**
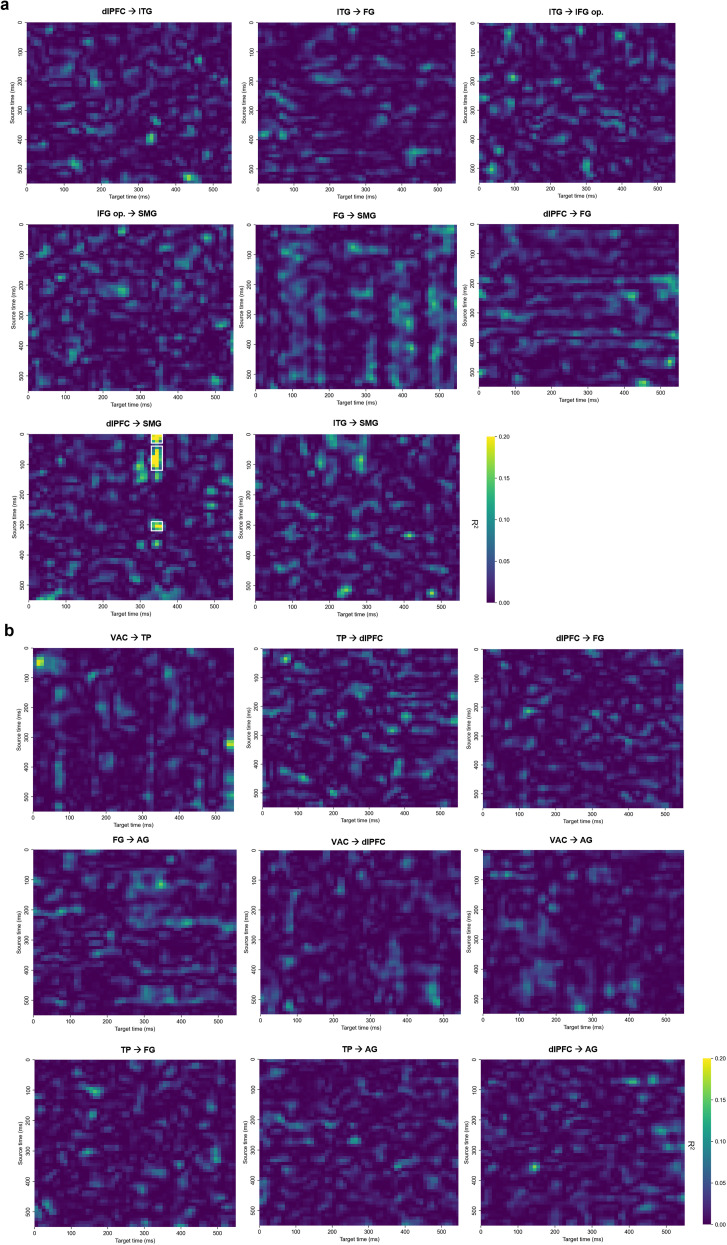
Cross-temporal linear regression results for body-part words. Each heat map shows the coefficient of determination (*R*^2^) for every source-time (*y*-axis) × target-time (*x*-axis) pair. Panels in ***a*** display left-hemisphere BA pairs, and panels in ***b*** display the corresponding right-hemisphere pairs; the panel titles specify the direction of prediction (source→target). For every pixel, the observed *R*^2^ was tested against a null distribution generated from 1,000 label shuffles, and the resulting *p* values were corrected with the Benjamini–Hochberg FDR procedure (*α* = 0.05). A cluster is highlighted with a white square only if all of its pixels both survived FDR correction and met the *R*^2^ threshold (seed ≥0.20, grown to 8-connected neighbors ≥0.10). Cross-temporal results for the combined-category analysis are provided in Extended Data Figure 7-1.

**Figure 8.**
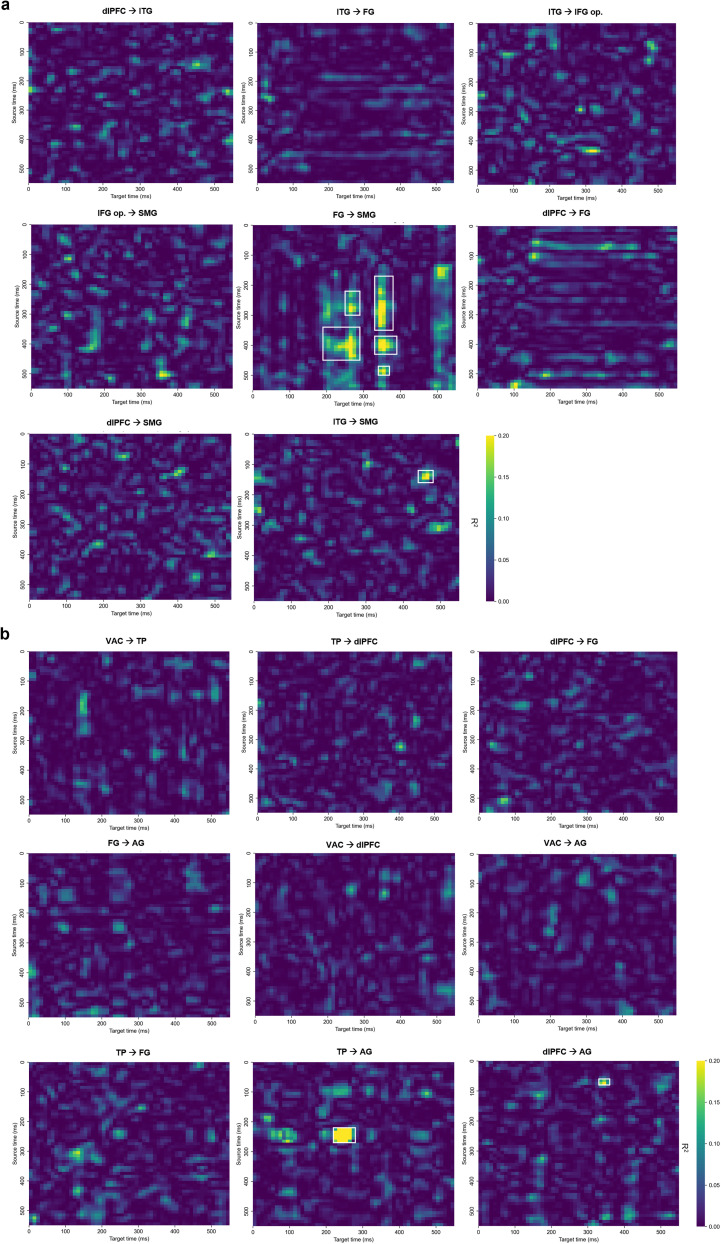
Cross-temporal linear regression results for nonbody words. Heat maps show *R*^2^ for each source-time (*y*) × target-time (*x*) pair: (***a***) left-hemisphere BA pairs, (***b***) right-hemisphere pairs. All matrices share a common color scale (*R*^2^ = 0–0.20), except for one cluster (TP→AG) that peaked at *R*^2^ = 0.70. *R*^2^ values were tested against 1,000 shuffle nulls and FDR-corrected (*α* = 0.05); only clusters whose pixels passed FDR and met the *R*^2^ threshold (seed ≥0.20, grown to 8-connected neighbors ≥0.10) are highlighted with a white square. Cross-temporal results for the combined-category analysis are provided in Extended Data Figure 8-1.

Cross-temporal linear regression matrices (Figs. 7, 8) illustrate how activity in an “early” (≤250 ms) BA predicts responses in distant regions over time. All matrices are displayed on a common color scale (*R*^2^ = 0–0.20), with the exception of a single cluster (right TP to angular gyrus) that exceeded this range, reaching a peak *R*^2^ of 0.70. All reported cells exceeded the 95th percentile of 1,000 permutation-based null distributions. Clusters were identified only when all constituent pixels both passed the Benjamini–Hochberg FDR correction (α = 0.05) and met the *R*^2^ threshold criteria—seed values ≥0.20 grown to eight-connected neighbors ≥0.10. Clusters smaller than 5 pixels were excluded, and each surviving cluster was enclosed with a white rectangle to indicate its full spatiotemporal extent in the source–target *R*^2^ matrix

10.1523/ENEURO.0254-25.2026.f7-1 Figure 7-1 Cross-temporal regression results for the combined-category analysis. Heat maps display R² values across source-time × target-time pairs for left hemisphere BA pairs. Only values exceeding the 95th percentile of 1,000 permutation-based null distributions are shown. Titles indicate source → target direction. Download Figure 7-1, TIF file.

10.1523/ENEURO.0254-25.2026.f8-1 Figure 8-1 Cross-temporal regression results for the combined-category analysis. Heat maps display R² values across source-time × target-time pairs for right hemisphere BA pairs. Only values exceeding the 95th percentile of 1,000 permutation-based null distributions are shown. Titles indicate source → target direction. Download Figure 8-1, TIF file.

Analyses of the pooled (combined-category) data revealed few but interpretable predictive relationships. No significant sustained cluster was found in the left hemisphere; however, in the right hemisphere, activity in the TP (200–300 ms) predicted angular gyrus responses at 200–250 ms (peak *R*^2^ = 0.61; Extended Data Figs. 7-1, 8-1).

Category-specific analyses revealed clearer and more sustained patterns of interregional prediction. For body–word trials, left dlPFC activity at 0–150 ms (peak *R*^2^ = 0.39) and again at 300–350 ms (peak *R*^2^ = 0.21) predicted SMG responses at 300–350 ms. For nonbody trials, in the left hemisphere, FG activity from 150 to 500 ms partially predicted SMG responses during 200–500 ms, with peak *R*^2^ values reaching 0.28. In the right hemisphere, a strong and temporally specific link was observed from the TP to the angular gyrus: TP activity at 200–300 ms predicted AG responses at 200–300 ms (peak *R*^2^ = 0.71)—the highest value across all tested pairs.

**Figure 9.**
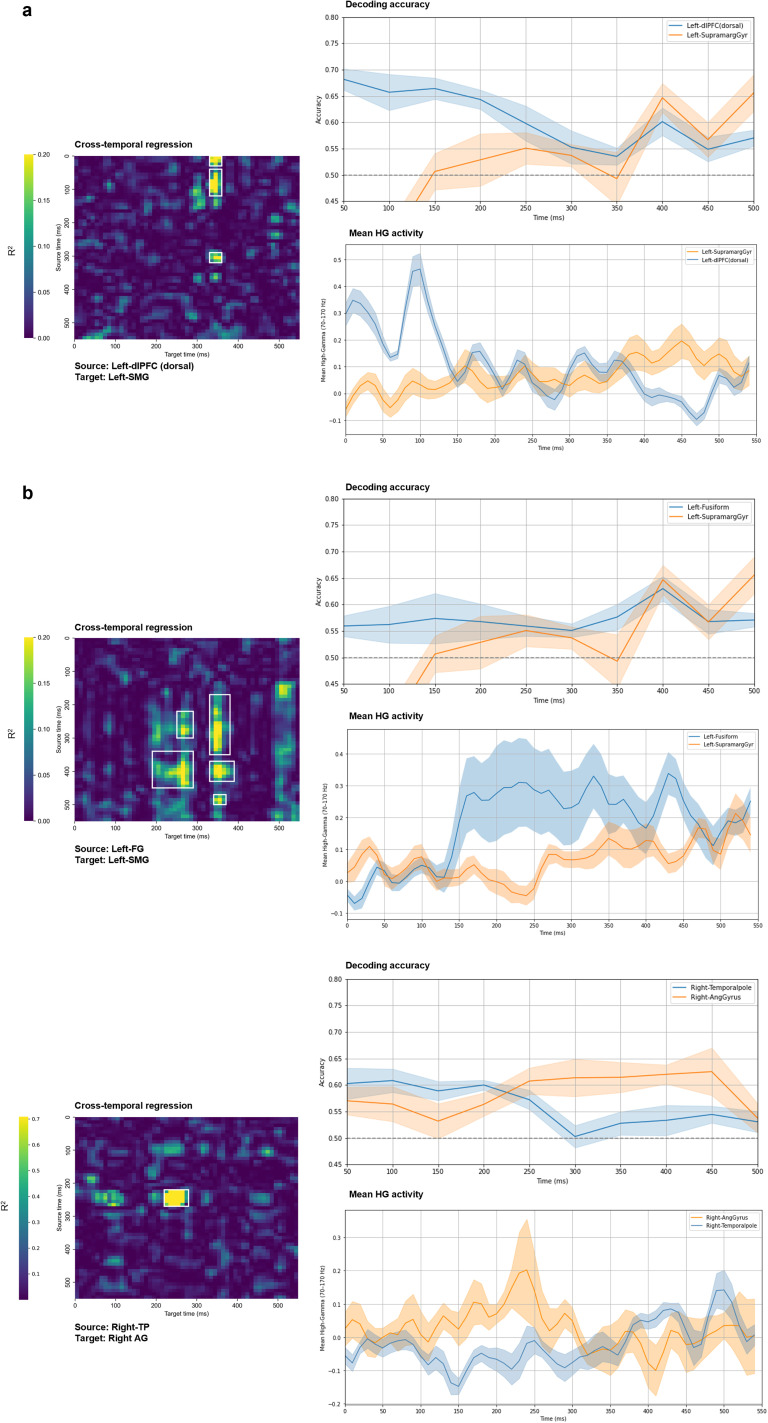
Decoding accuracy traces and mean HG activity for each source–target BA pair with significant cross-temporal regression. Traces were extracted from Figure 5, restricted to the BA pairs identified in the regression analysis. ***a***, Body category (left dlPFC→SMG). ***b***, Nonbody category (left fusiform→SMG; right TP→AG). These comparisons illustrate consistent temporal offsets across decoding accuracy, HG activity, and regression patterns.

To further validate the directional relationships observed in the regression matrices, we plotted decoding accuracy and mean HG activity for each significant source–target BA pair (Fig. 9). These traces were drawn from the time-resolved classification results (Fig. 5), restricted to the relevant regions identified in the cross-temporal analysis. By aligning classification performance and raw signal profiles with regression outcomes, this visualization offers converging evidence for temporally structured interregional dynamics during prespeech semantic processing. In the body condition (Fig. 9*a*), early HG activation and elevated decoding in the left dlPFC preceded a rise in supramarginal decoding, reflecting the top–down influence captured in the regression map. In the nonbody condition (Fig. 9*b*), left fusiform activity led supramarginal responses in both HG amplitude and decoding, supporting its predictive role. The right TP and angular gyrus pair similarly showed aligned patterns across all three measures, reinforcing the robustness of their temporally specific interaction.

## Discussion

From this study, semantic representations were decoded during the prespeech period. HG ECoG activity enabled reliable classification of body-part versus nonbody-part words, with performance substantially above chance. By revealing the timing and anatomical distribution of conceptual information prior to articulation, these findings advance understanding of higher-level language processing beyond motor or acoustic output and inform potential applications for BCI systems.

The results indicate that semantic decoding is not limited to isolated cortical loci but arises through coordinated engagement of different regions over time. This dynamic perspective challenges models that emphasize static or uniform representations and underscores that prespeech conceptual processing involves a temporally structured cascade of neural activity.

### Time-resolved semantic decoding and bilateral engagement

Semantic category information was decodable from HG activity with peak accuracies emerging across both hemispheres. Notably, the maximum accuracy of 68% observed here exceeds the performance typically reported in semantic decoding studies focusing on group-level binary classification, where mean accuracies are only modestly above chance, approximately between 50 and 60% (Simanova et al., 2015; Magnabosco and Hauk, 2024). The left hemisphere exhibited a sequential cascade, beginning in the dlPFC and progressing through the ITG, FG, and finally the SMG, suggesting a frontal–temporal–occipital–parietal engagement. In contrast, the right hemisphere exhibited a distinct occipital–temporal–frontal–occipital–parietal progression, with early engagement of the VAC and TP, followed by the dlPFC, FG, and angular gyrus. Although some occipitotemporal electrodes overlapped with the extrastriate body area (EBA; Extended Data Table 3-1), which is primarily involved in visual body perception (Downing et al., 2001; Urgesi et al., 2007), the decoding effects observed here are better interpreted as reflecting semantic access to visually grounded concepts from written words rather than perceptual body processing.

These asymmetric trajectories suggest that the hemispheres contribute differently to prespeech semantic processing—not via mirrored bilateral engagement as proposed in the dual-stream model (Hickok and Poeppel, 2007) but via a temporally staggered division of labor. The left hemisphere may initiate early semantic integration, while the right supports complementary or domain-general processes (Jung-Beeman, 2005; Vigneau et al., 2006; Mech et al., 2022). However, it remains possible that some right-hemisphere decoding reflects transcallosal projections (Bloom and Hynd, 2005) or detection of left-hemisphere signals via contralateral electrodes, and future studies with bilateral coverage will be necessary to clarify these contributions. Importantly, the left-hemisphere regional and temporal pattern of peak decoding remained consistent after inclusion of an additional subject analyzed with identical preprocessing and statistical procedures (Extended Data Fig. 5-4).

### Predictive cortical interactions prior to speech onset

Beyond identifying where semantic information is most decodable, cross-temporal linear regression uncovered how activity in early time windows predicts subsequent responses in anatomically distant regions. This analysis reveals directional temporal dependencies that extend our understanding of how distributed cortical areas coordinate semantic category processing during the prespeech period. We selected this approach because it provides time-resolved estimates of directional dependencies across all source and target-time pairs, allows direct quantification of explained variance in the form of interpretable *R*^2^ values, and is robust to high-dimensional ECoG data without requiring the large sample sizes and discretization steps that information–theoretic measures often entail. While alternative methods such as Granger causality can be used to assess directional influence, they typically summarize predictive influence as a single statistic over the time series (Seth et al., 2015), whereas cross-temporal regression provides time-resolved estimates between all pairs of time windows.

For the body-part category, left dlPFC activity at both 0–150 ms and 300–350 ms significantly predicted SMG responses at 300–350 ms. This suggests an early and possibly sustained top–down influence from frontal executive regions toward parietal integration hubs, supporting the view that semantic category processing recruits coordinated control mechanisms prior to speech initiation. Such dynamics are consistent with the “Control” operations described in Hagoort's Memory-Unification-Control (MUC) framework (Hagoort, 2013).

For the nonbody-part category, a strong and temporally precise relationship was observed from the right TP to the right angular gyrus, at 200–300 ms. Early TP activity may reflect rapid presemantic orienting processes that precede the emergence of stable conceptual representations (Farahibozorg et al., 2022). The cross-temporal analyses indicated that predictive influences became most robust during 200–300 ms, linking TP activity to angular gyrus responses at the same latency. The peak *R*^2^ value of 0.71 was the highest among all tested source–target pairs. Additionally, left FG activity from 150 to 500 ms predicted SMG responses during 200–500 ms, further supporting the role of parietal regions as integrative convergence zones. This pattern is consistent with accounts proposing that anterior temporal regions provide rapid inputs that are subsequently integrated within parietal semantic hubs (Farahibozorg et al., 2022), the FG's involvement in semantic feature binding (Martin, 2007), and the angular gyrus as a site of semantic convergence (Patterson et al., 2007; Binder et al., 2009). Anatomical evidence from diffusion tractography and histological studies further demonstrates that the middle longitudinal fasciculus connects the TP and superior temporal regions to the angular gyrus (Makris et al., 2013; Kalyvas et al., 2020). The temporally precise predictive dynamics observed here likely reflect interregional engagement along this pathway, with anterior–temporal activity providing early access signals that feed into parietal regions where semantic meaning becomes activated.

Crucially, across both semantic categories, all significant predictive target regions were located in the parietal lobe—the supramarginal and angular gyri. This suggests a convergent role for the parietal cortex in integrating upstream information before speech output. Such parietal involvement aligns with theories emphasizing its function in multimodal semantic representation and combinatorial meaning assembly (Binder and Desai, 2011; Seghier, 2013). Notably, the source regions differed by semantic category. Nonbody words may rely more on orthographic-to-semantic mapping and the retrieval of visually grounded concepts, processes supported by the FG (Price and Devlin, 2011; De¸bska et al., 2023), whereas body-related words are more strongly associated with action and embodied representations, requiring controlled retrieval and top–down integration mediated by the DLPFC (Moseley et al., 2012; Hagoort, 2013; Hertrich et al., 2021).

These results provide evidence that temporally structured interregional interactions, culminating in parietal targets, form a key component of prespeech semantic processing—highlighting how the brain dynamically organizes categorical information in preparation for language production.

### Revisiting language models with dynamic prespeech evidence

To place these findings in the context of existing theories, we compared our results with established models of language processing. While classical models of language processing have outlined the anatomy and timing of semantic integration, our decoding results challenge several of their core assumptions. For instance, Friederici's hierarchical model proposes a bottom–up sequence from posterior to anterior regions (Friederici, 2011), yet our peak decoding often emerged earliest in frontal cortices. Similarly, the dual-stream model posits symmetrical, parallel processing across hemispheres (Hickok and Poeppel, 2007), whereas we observed temporally offset and functionally asymmetric decoding dynamics. Hagoort's MUC model, though accounting for top–down control, does not specify the temporal or predictive relationships we identified (Hagoort, 2013).

By combining classification and regression within the prespeech window, we move beyond static activation maps and offer a dynamic view of how semantic categories are encoded and transmitted between regions. These findings refine existing models by demonstrating how temporally organized neural interactions contribute to conceptual processing before articulation.

### Implications and future directions

While this study demonstrates that HG activity during the prespeech period can support reliable decoding of semantic categories and reveal temporally structured predictive interactions across cortical regions, several limitations should be considered. First, each subject completed only a single presentation of each word, yielding 34 trials in total. This relatively small number of observations may have reduced statistical power and helps explain the modest decoding accuracies. However, the combination of cross-validation, permutation testing, and FDR correction ensured that the reported results reflect statistically reliable decoding despite this constraint. Second, our analyses were restricted to single-word stimuli without contextual or syntactic information, which limits generalization to more naturalistic language use. Additionally, electrode coverage did not include bilateral or subcortical regions, precluding assessment of interhemispheric dynamics and deeper network contributions. Future studies incorporating sentence-level tasks and depth recordings could provide a more comprehensive perspective on how conceptual information is integrated across broader language networks (Piai et al., 2016). Moreover, the vocabulary set, though validated for acoustic decoding (Meng et al., 2023), represented a constrained range of semantic content. Although major psycholinguistic properties were matched, body-part words were more concrete than nonbody words (Extended Data Fig. 2-3). This difference may contribute to decoding but is closely aligned with the intrinsic semantic properties of body-part concepts (Barsalou, 2008). At the methodological level, future work could also consider refining the grouping strategy, for example, by subdividing cortical regions and aggregating signals from spatially adjacent channels, which may enhance sensitivity while reducing the influence of noninformative sites.

Despite these constraints, the present findings highlight the feasibility of identifying intention-level conceptual representations before articulation begins. By delineating when and where semantic information is expressed and how it propagates across cortical regions, this work contributes to a more dynamic understanding of language processing. In the longer term, such insights could inform the development of BCI strategies that aim to decode internally generated concepts, although further research will be necessary to establish their practical implementation. Together, these results help bridge fundamental neurocognitive models with emerging applications in neural decoding.

## Synthesis

Reviewing Editor: Adrien Peyrache, McGill University Decisions are customarily a result of the Reviewing Editor and the peer reviewers coming together and discussing their recommendations until a consensus is reached. When revisions are invited, a fact-based synthesis statement explaining their decision and outlining what is needed to prepare a revision will be listed below. The following reviewer(s) agreed to reveal their identity: NONE.

The authors have addressed the reviewers' comments. The manuscript is now suitable for publication.

## References

[B1] Akbari H, Khalighinejad B, Herrero JL, Mehta AD, Mesgarani N (2019) Towards reconstructing intelligible speech from the human auditory cortex. Sci Rep 9:874. 10.1038/s41598-018-37359-z 30696881 PMC6351601

[B2] Barsalou LW (2008) Grounded cognition. Annu Rev Psychol 59:617–645. 10.1146/annurev.psych.59.103006.093639 17705682

[B3] Binder JR, Desai RH, Graves WW, Conant LL (2009) Where is the semantic system? A critical review and meta-analysis of 120 functional neuroimaging studies. Cereb Cortex 19:2767–2796. 10.1093/cercor/bhp055 19329570 PMC2774390

[B4] Binder JR, Desai RH (2011) The neurobiology of semantic memory. Trends Cogn Sci 15:527–536. 10.1016/j.tics.2011.10.001 22001867 PMC3350748

[B5] Bloom JS, Hynd GW (2005) The role of the corpus callosum in interhemispheric transfer of information: excitation or inhibition? Neuropsychol Rev 15:59–71. 10.1007/s11065-005-6252-y 16211466

[B6] Brouwer H, Crocker MW, Venhuizen NJ, Hoeks JC (2017) A neurocomputational model of the N400 and the P600 in language processing. Cogn Sci 41:1318–1352. 10.1111/cogs.12461 28000963 PMC5484319

[B7] Brown C, Hagoort P (1993) The processing nature of the N400: evidence from masked priming. J Cogn Neurosci 5:34–44. 10.1162/jocn.1993.5.1.34 23972118

[B8] Brumberg JS, Wright EJ, Guenther FH, Kennedy PR (2011) Classification of intended phoneme production from chronic intracortical microelectrode recordings in speech motor cortex. Front Neurosci 5:7880. 10.3389/fnins.2011.00065 PMC3096823 21629876

[B9] Crone NE, Sinai A, Korzeniewska A (2006) High-frequency gamma oscillations and human brain mapping with electrocorticography. Prog Brain Res 159:275–295. 10.1016/S0079-6123(06)59019-3 17071238

[B10] De¸bska A, Wójcik M, Chyl K, Dzie¸giel-Fivet G, Jednoróg K (2023) Beyond the visual word form area–a cognitive characterization of the left ventral occipitotemporal cortex. Front Hum Neurosci 17:1199366. 10.3389/fnhum.2023.1199366 37576470 PMC10416454

[B11] Dichter BK, Breshears JD, Leonard MK, Chang EF (2018) The control of vocal pitch in human laryngeal motor cortex. Cell 174:21–31.e29. 10.1016/j.cell.2018.05.016 29958109 PMC6084806

[B12] Downing PE, Jiang Y, Shuman M, Kanwisher N (2001) A cortical area selective for visual processing of the human body. Science 293:2470–2473. 10.1126/science.1063414 11577239

[B13] Duffy EI, Garry J, Talbot L, Pasternak D, Flinn A, Minardi C, Dookram M, Grant K, Fitzgerald D, Rubano J (2018) A pilot study assessing the spiritual, emotional, physical/environmental, and physiological needs of mechanically ventilated surgical intensive care unit patients via eye tracking devices, head nodding, and communication boards. Trauma Surg Acute Care Open 3:e000180. 10.1136/tsaco-2018-000180 30246152 PMC6144907

[B14] Falach R, et al. (2024) Annotated interictal discharges in intracranial EEG sleep data and related machine learning detection scheme. Sci Data 11:1354. 10.1038/s41597-024-04187-y 39695255 PMC11655530

[B15] Farahibozorg S-R, Henson RN, Woollams AM, Hauk O (2022) Distinct roles for the anterior temporal lobe and angular gyrus in the spatiotemporal cortical semantic network. Cereb Cortex 32:4549–4564. 10.1093/cercor/bhab501 35094061 PMC9574238

[B16] Friederici AD (2011) The brain basis of language processing: from structure to function. Physiol Rev 91:1357. 10.1152/physrev.00006.2011 22013214

[B17] Hagoort P (2013) MUC (memory, unification, control) and beyond. Front Psychol 4:416. 10.3389/fpsyg.2013.00416 23874313 PMC3709422

[B18] Hagoort P (2017) The core and beyond in the language-ready brain. Neurosci Biobehav Rev 81:194–204. 10.1016/j.neubiorev.2017.01.048 28193452

[B19] Hertrich I, Dietrich S, Blum C, Ackermann H (2021) The role of the dorsolateral prefrontal cortex for speech and language processing. Front Hum Neurosci 15:645209. 10.3389/fnhum.2021.645209 34079444 PMC8165195

[B20] Hickok G, Poeppel D (2007) The cortical organization of speech processing. Nat Rev Neurosci 8:393–402. 10.1038/nrn2113 17431404

[B21] Huth AG, De Heer WA, Griffiths TL, Theunissen FE, Gallant JL (2016) Natural speech reveals the semantic maps that tile human cerebral cortex. Nature 532:453–458. 10.1038/nature17637 27121839 PMC4852309

[B22] Johnson E, Bornman J, Tönsing KM (2016) An exploration of pain-related vocabulary: implications for AAC use with children. Augment Altern Commun 32:249–260. 10.1080/07434618.2016.1233998 27712115

[B23] Jung-Beeman M (2005) Bilateral brain processes for comprehending natural language. Trends Cogn Sci 9:512–518. 10.1016/j.tics.2005.09.009 16214387

[B24] Kalyvas A, Koutsarnakis C, Komaitis S, Karavasilis E, Christidi F, Skandalakis GP, Liouta E, Papakonstantinou O, Kelekis N, Duffau H (2020) Mapping the human middle longitudinal fasciculus through a focused anatomo-imaging study: shifting the paradigm of its segmentation and connectivity pattern. Brain Struct Funct 225:85–119. 10.1007/s00429-019-01987-6 31773331

[B25] Kutas M, Federmeier KD (2011) Thirty years and counting: finding meaning in the N400 component of the event-related brain potential (ERP). Annu Rev Psychol 62:621–647. 10.1146/annurev.psych.093008.131123 20809790 PMC4052444

[B26] Lacadie C, Fulbright R, Arora J, Constable R, Papademetris X (2008) Brodmann areas defined in MNI space using a new Tracing Tool in BioImage Suite. In: Proceedings of the 14th Annual Meeting of the Organization for Human Brain Mapping.

[B27] Magnabosco F, Hauk O (2024) Decoding semantics from dynamic brain activation patterns: from trials to task in EEG/MEG source space. eNeuro 11:ENEURO.0277-23.2023. 10.1523/ENEURO.0277-23.2023 PMC10913025 38320767

[B28] Makris N, Preti M, Wassermann D, Rathi Y, Papadimitriou G, Yergatian C, Dickerson BC, Shenton ME, Kubicki M (2013) Human middle longitudinal fascicle: segregation and behavioral-clinical implications of two distinct fiber connections linking temporal pole and superior temporal gyrus with the angular gyrus or superior parietal lobule using multi-tensor tractography. Brain Imaging Behav 7:335–352. 10.1007/s11682-013-9235-2 23686576 PMC3830590

[B29] Martin A (2007) The representation of object concepts in the brain. Annu Rev Psychol 58:25–45. 10.1146/annurev.psych.57.102904.190143 16968210

[B30] Mazurchuk S, Fernandino L, Tong J-Q, Conant LL, Binder JR (2024) The neural representation of body part concepts. Cereb Cortex 34:bhae213. 10.1093/cercor/bhae213 38863113 PMC11166504

[B31] Mech EN, Kandhadai P, Federmeier KD (2022) The last course of coarse coding: hemispheric similarities in associative and categorical semantic processing. Brain Lang 229:105123. 10.1016/j.bandl.2022.105123 35461030 PMC9214668

[B32] Meng K, Goodarzy F, Kim E, Park YJ, Kim JS, Cook MJ, Chung CK, Grayden DB (2023) Continuous synthesis of artificial speech sounds from human cortical surface recordings during silent speech production. J Neural Eng 20:046019. 10.1088/1741-2552/ace7f6 37459853

[B33] Moseley R, Carota F, Hauk O, Mohr B, Pulvermüller F (2012) A role for the motor system in binding abstract emotional meaning. Cereb Cortex 22:1634–1647. 10.1093/cercor/bhr238 21914634 PMC3377965

[B34] Moses DA, Leonard MK, Makin JG, Chang EF (2019) Real-time decoding of question-and-answer speech dialogue using human cortical activity. Nat Commun 10:3096. 10.1038/s41467-019-10994-4 31363096 PMC6667454

[B35] Mugler EM, Patton JL, Flint RD, Wright ZA, Schuele SU, Rosenow J, Shih JJ, Krusienski DJ, Slutzky MW (2014) Direct classification of all American English phonemes using signals from functional speech motor cortex. J Neural Eng 11:035015. 10.1088/1741-2560/11/3/035015 24836588 PMC4097188

[B36] Nagata K, Kunii N, Shimada S, Fujitani S, Takasago M, Saito N (2022) Spatiotemporal target selection for intracranial neural decoding of abstract and concrete semantics. Cereb Cortex 32:5544–5554. 10.1093/cercor/bhac034 35169837 PMC9753048

[B37] Patterson K, Nestor PJ, Rogers TT (2007) Where do you know what you know? The representation of semantic knowledge in the human brain. Nat Rev Neurosci 8:976–987. 10.1038/nrn2277 18026167

[B38] Piai V, Anderson KL, Lin JJ, Dewar C, Parvizi J, Dronkers NF, Knight RT (2016) Direct brain recordings reveal hippocampal rhythm underpinnings of language processing. Proc Natl Acad Sci U S A 113:11366–11371. 10.1073/pnas.1603312113 27647880 PMC5056038

[B39] Price CJ, Devlin JT (2011) The interactive account of ventral occipitotemporal contributions to reading. Trends Cogn Sci 15:246–253. 10.1016/j.tics.2011.04.001 21549634 PMC3223525

[B40] Proix T, Delgado Saa J, Christen A, Martin S, Pasley BN, Knight RT, Tian X, Poeppel D, Doyle WK, Devinsky O (2022) Imagined speech can be decoded from low-and cross-frequency intracranial EEG features. Nat Commun 13:48. 10.1038/s41467-021-27725-3 35013268 PMC8748882

[B41] Schönmann I, Szewczyk J, de Lange FP, Heilbron M (2025) “Stimulus dependencies—rather than next-word prediction—can explain pre-onset brain encoding during natural listening.” bioRxiv: 2025.2003. 2008.642140. 10.7554/eLife.106543 PMC13068430 41960890

[B42] Seghier ML (2013) The angular gyrus: multiple functions and multiple subdivisions. Neuroscientist 19:43–61. 10.1177/1073858412440596 22547530 PMC4107834

[B43] Seth AK, Barrett AB, Barnett L (2015) Granger causality analysis in neuroscience and neuroimaging. J Neurosci 35:3293–3297. 10.1523/JNEUROSCI.4399-14.2015 25716830 PMC4339347

[B44] Simanova I, Van Gerven M, Oostenveld R, Hagoort P (2010) Identifying object categories from event-related EEG: toward decoding of conceptual representations. PLoS One 5:e14465. 10.1371/journal.pone.0014465 21209937 PMC3012689

[B45] Simanova I, Van Gerven MA, Oostenveld R, Hagoort P (2015) Predicting the semantic category of internally generated words from neuromagnetic recordings. J Cogn Neurosci 27:35–45. 10.1162/jocn_a_00690 25061927

[B46] Urgesi C, Candidi M, Ionta S, Aglioti SM (2007) Representation of body identity and body actions in extrastriate body area and ventral premotor cortex. Nat Neurosci 10:30–31. 10.1038/nn1815 17159990

[B47] Vigneau M, Beaucousin V, Hervé P-Y, Duffau H, Crivello F, Houde O, Mazoyer B, Tzourio-Mazoyer N (2006) Meta-analyzing left hemisphere language areas: phonology, semantics, and sentence processing. Neuroimage 30:1414–1432. 10.1016/j.neuroimage.2005.11.002 16413796

[B48] Xia M, Wang J, He Y (2013) Brainnet viewer: a network visualization tool for human brain connectomics. PLoS One 8:e68910. 10.1371/journal.pone.0068910 23861951 PMC3701683

[B49] Xu Y, He Y, Bi Y (2017) A tri-network model of human semantic processing. Front Psychol 8:1538. 10.3389/fpsyg.2017.01538 28955266 PMC5600905

[B50] Zhang D, Wang Z, Qian Y, Zhao Z, Liu Y, Lu J, Li Y (2025) Protocol to perform offline ECoG brain-to-text decoding for natural tonal sentences. STAR Protoc 6:103650. 10.1016/j.xpro.2025.103650 39985774 PMC11904493

[B51] Zhao C, Liu Y, Zeng J, Luo X, Sun W, Luan G, Liu Y, Zhang Y, Shi G, Guan Y (2024) Spatiotemporal neural network for sublexical information processing: an intracranial SEEG study. J Neurosci 44:e0717242024. 10.1523/JNEUROSCI.0717-24.2024 39214706 PMC11551892

