# Supplementary material for: Spatiotemporal Dynamics in Prespeech Semantic Category Decoding: An Intracranial EEG Study
Source: eNeuro. 2026 Apr 21;13(4):ENEURO.0254-25.2026. doi: 10.1523/ENEURO.0254-25.2026 (PMC13116012; doi:10.1523/ENEURO.0254-25.2026)
Supplement: Figure 2-1 — A categorized list of 34 words (body-part vs. non-body-part) was used in the speech-production task; the words were randomly displayed on the screen one at a time. Download Figure 2-1, DOCX file. [file eneuro-13-ENEURO.0254-25.2026-s003.docx]

**Figure 2-1.**

| **Body part** | **Non-body part** |
| --- | --- |
| 손;hand  머리;head  팔;arm  귀;ear  눈;eye  코;nose  입;mouth  얼굴;face  뼈;bone  피부;skin  등;back  허리;waist  목;neck  가슴;chest  배;stomach  발;foot  다리;leg | 하나;one  둘;two  셋;three  넷;four  다섯;five  여섯;six  일곱;seven  여덟;eight  아홉;nine  열;ten  물;water  음식;food  텔레비전;television  집;home  침대;bed  병원;hospital  화장실;toilet |
