# Supplementary figures and images for: Spatiotemporal Dynamics in Prespeech Semantic Category Decoding: An Intracranial EEG Study
Source: eNeuro. 2026 Apr 21;13(4):ENEURO.0254-25.2026. doi: 10.1523/ENEURO.0254-25.2026 (PMC13116012; doi:10.1523/ENEURO.0254-25.2026)

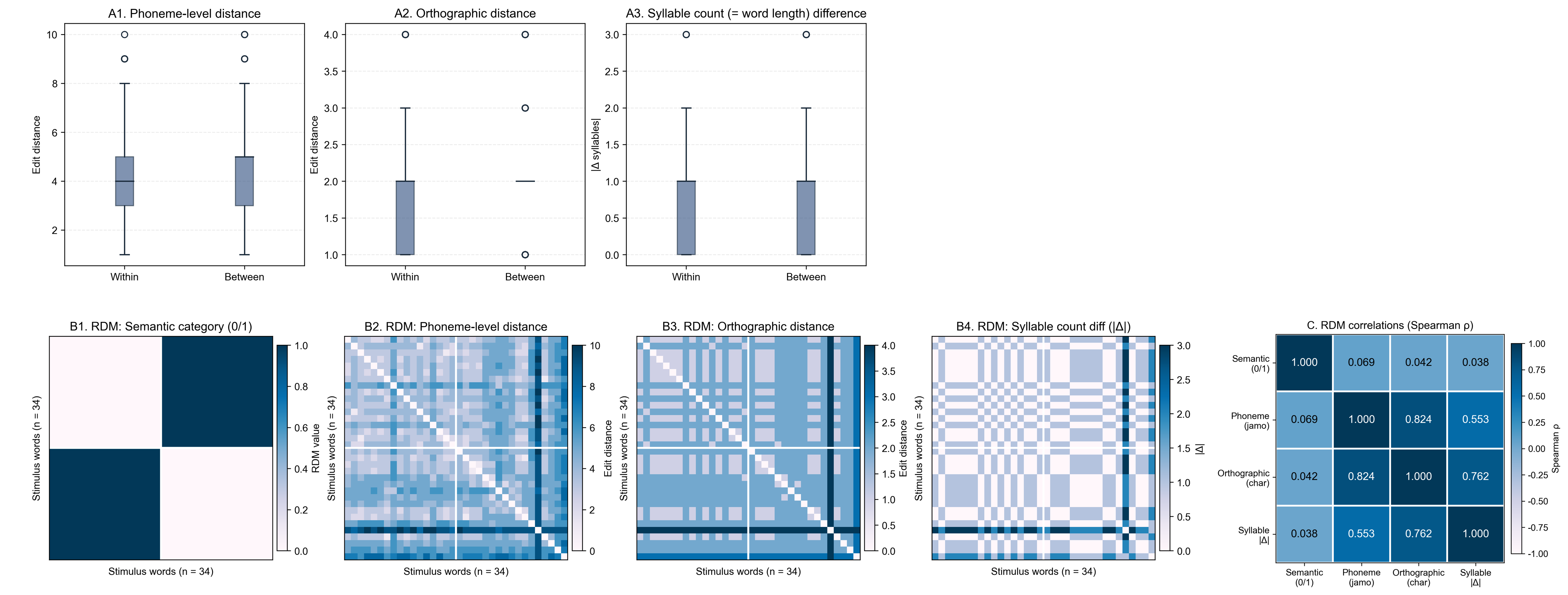

Supplement: Figure 2-2 — Control analyses of low-level structural properties across stimulus words. Phoneme-level distance and syllable count differences were examined to assess potential low-level confounds. Phoneme-level distance was computed by decomposing each word into Korean Hangul phoneme (jamo) sequences and calculating the Levenshtein distance between sequence. Orthographic distance was computed as the Levenshtein edit distance between Hangul syllable characters, and syllable difference was defined as the absolute difference in syllable counts (|Δ|) between word pairs. (A) Pairwise comparisons between within- and between-category word pairs show no systematic increase in phoneme-level distance, orthographic distance, or syllable differences across semantic categories. (B) Representational dissimilarity matrices (RDMs) constructed from semantic category labels (0 = within-category, 1 = between-category), phoneme-level distance, orthographic distance, and syllable count differences reveal a clear categorical block structure only in the semantic RDM, whereas the phoneme-level, orthographic, and syllable RDMs do not exhibit corresponding organization. (C) Spearman correlations computed across all unique pairwise dissimilarity values indicate minimal association between semantic category structure and phoneme-level distance (ρ = 0.069), orthographic distance (ρ = 0.042) or syllable difference (ρ = 0.038), while phoneme-level distance and syllable difference show a moderate correlation (ρ = 0.553). These results indicate that the semantic categorical structure is not accounted for by the measured low-level phonological or syllabic properties. Download Figure 2-2, TIF file. [file eneuro-13-ENEURO.0254-25.2026-s004.tif]

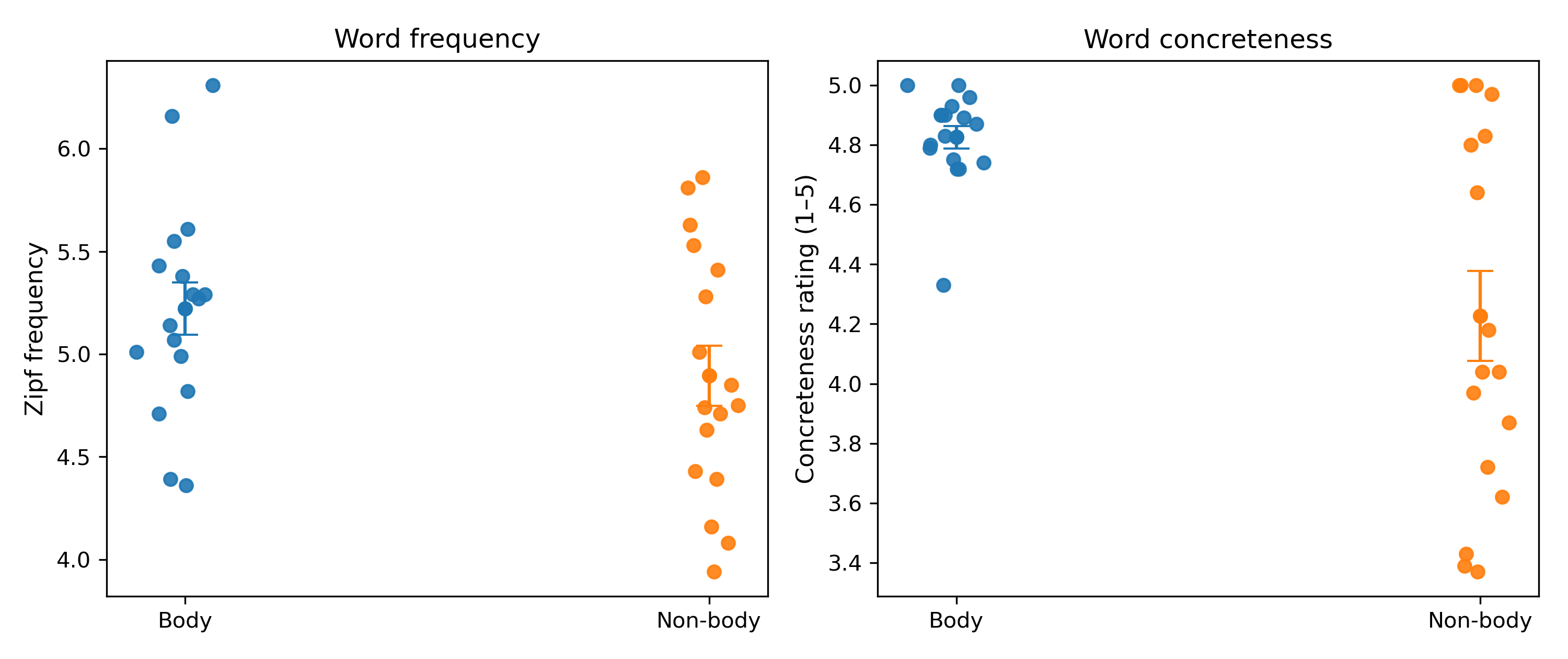

Supplement: Figure 2-3 — Psycholinguistic comparison of stimulus sets. Scatter plots show word frequency (Zipf scale, Korean wordfreq norms; left) and concreteness ratings (English norms via translation; right) for body-part and non-body words. Word frequency did not differ significantly between categories (Welch’s p = 0.10; Mann–Whitney p = 0.15), whereas concreteness was significantly higher for body-part words (Welch’s p = 0.001; Mann–Whitney p = 0.023; Cohen’s d = 1.32). Error bars indicate mean ± standard error. Download Figure 2-3, TIF file. [file eneuro-13-ENEURO.0254-25.2026-s005.tif]

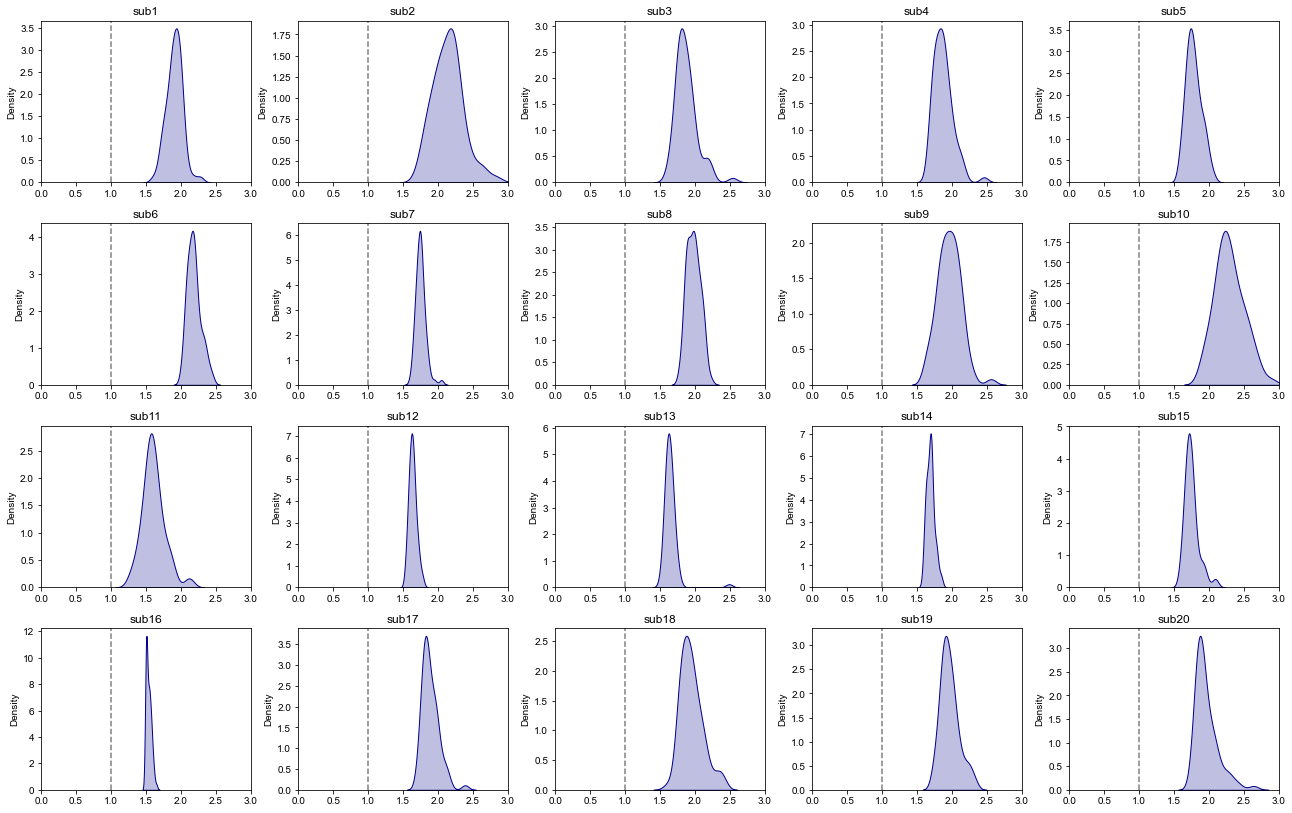

Supplement: Figure 3-1 — Kernel density estimates of speech-onset times for each subject. Time is referenced to fixation onset (0–3 s), with the dashed vertical line at 1 s marking word presentation. Density peaks across subjects typically fall between 1.5 and 2.5 s, corresponding to 0.5–1.5 s post-word onset. Subject 11 was excluded from further analyses due to a speech-onset distribution exceeding 2 SD from the group mean. Download Figure 3-1, TIF file. [file eneuro-13-ENEURO.0254-25.2026-s006.tif]

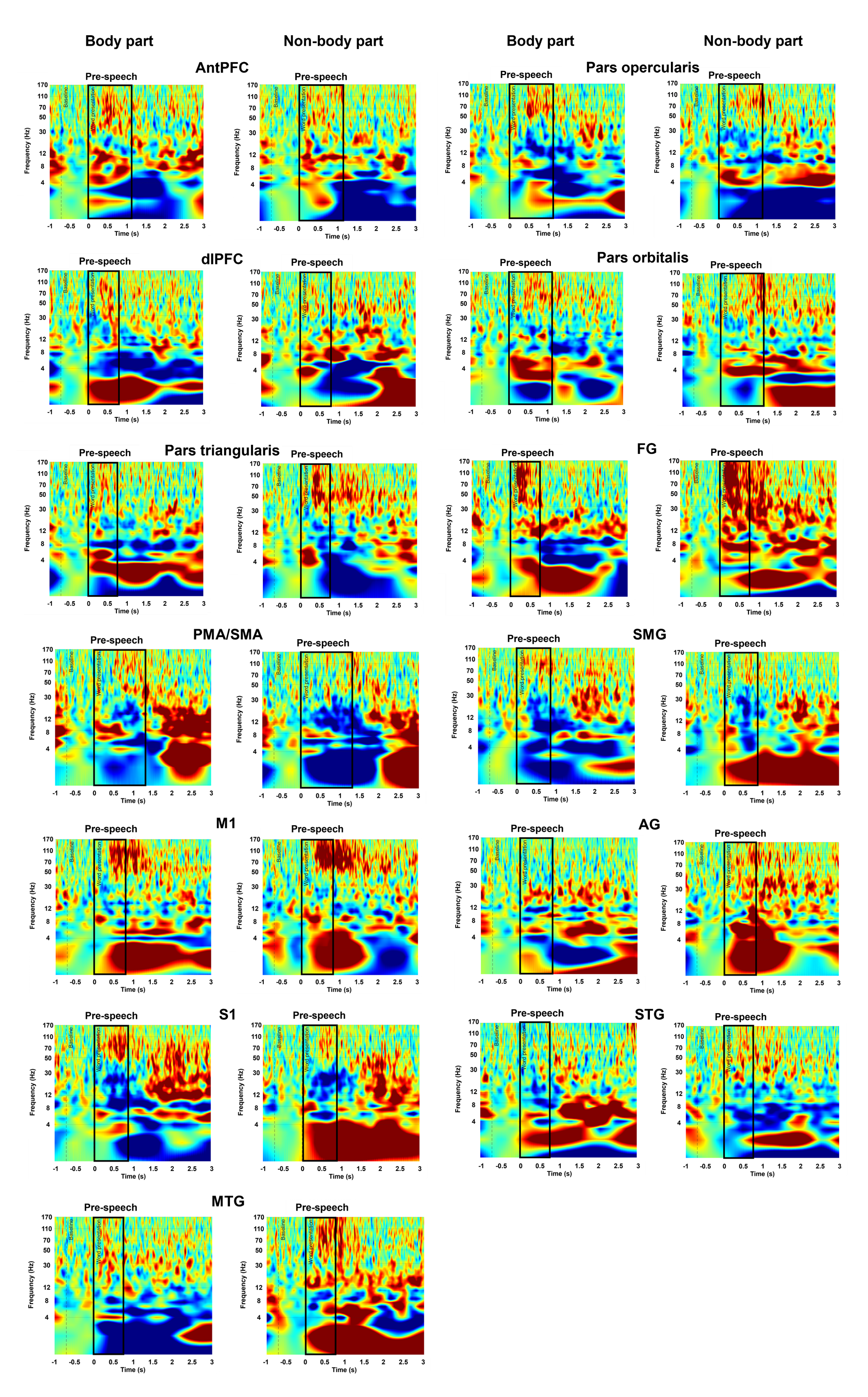

Supplement: Figure 4-1 — Time-frequency analysis showing spectral dynamics between the two semantic categories (body part vs. non-body part) during the word reading task, performed using continuous wavelet transform analysis. The period from -1 s to 0 s represents fixation, -0.3 s to 0 s serves as the baseline, and 0 s to 3 s represents the word presentation. The black square indicates the pre-speech period. This example is from an individual subject with a left hemisphere implant. Download Figure 4-1, TIF file. [file eneuro-13-ENEURO.0254-25.2026-s007.tif]

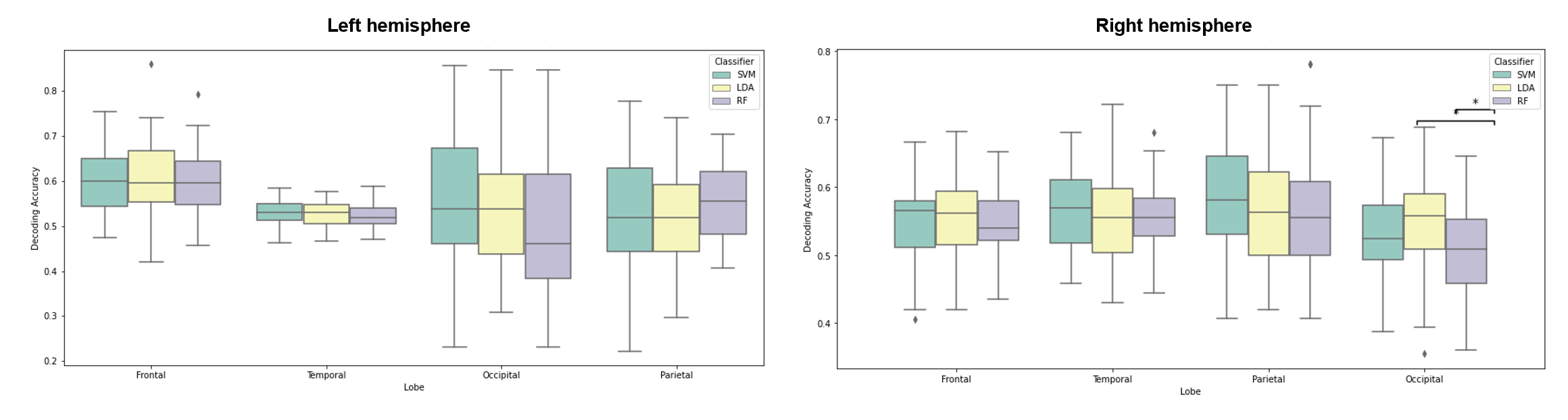

Supplement: Figure 5-1 — Box-plot comparison of decoding accuracy (averaged over 0–500 ms post-stimulus) across classifiers—Support Vector Machine (SVM), Linear Discriminant Analysis (LDA), and Random Forest (RF)—for the best-performing BA within each lobe. Left and right panels show results from the left and right hemispheres, respectively. Overall, decoding performance was comparable across classifiers, with no statistically significant differences observed in most lobes. However, in the right hemisphere's Occipital lobe, both SVM and LDA achieved significantly higher accuracy than RF (FDR-corrected p = 0.0136 and 0.0107, respectively). No other classifier pairwise comparisons reached significance following FDR correction (Asterisks indicate significant pairwise differences between classifiers (p < 0.05, FDR-corrected, within-lobe). Download Figure 5-1, TIF file. [file eneuro-13-ENEURO.0254-25.2026-s008.tif]

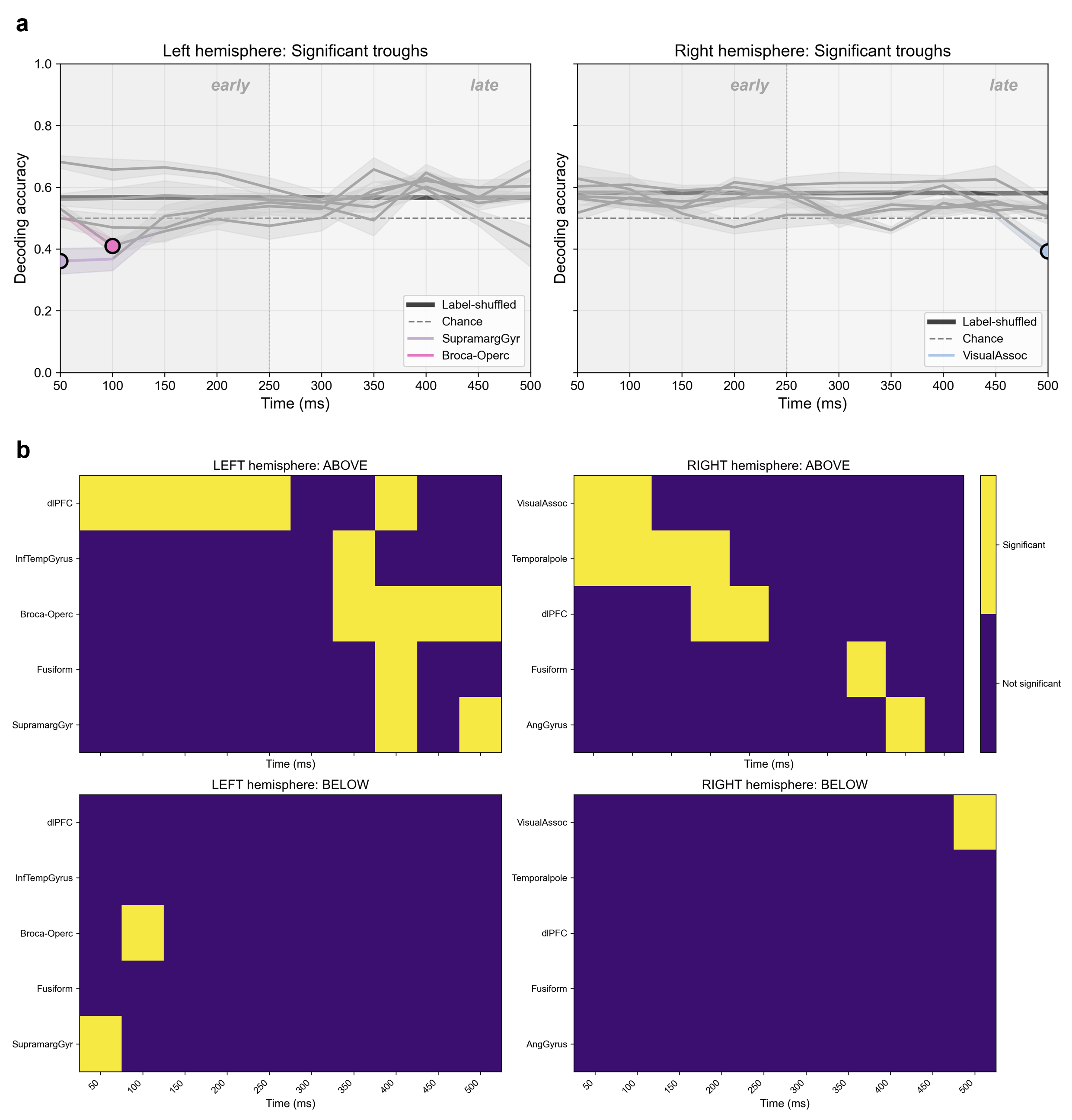

Supplement: Figure 5-2 — (A) Significant decoding troughs below chance level. Statistically significant decoding minima are shown for each Brodmann area (BA) in the left and right hemispheres. Minima were evaluated against the lower tail of the 2000-iteration label-shuffled permutation distribution with FDR correction across time windows within each BA, and only time points that also passed the time-shuffle threshold are displayed. Circles indicate significant troughs. (B) Asymmetric temporal distribution of significant decoding above and below chance. Binary matrices display time windows showing statistically significant decoding above (top panels) and below (bottom panels) chance for each BA in the left and right hemispheres. Identical statistical criteria were applied in both directions (2000-iteration label-shuffled permutation with FDR correction across time windows within each BA, combined with the time-shuffle threshold). Yellow cells indicate time points meeting both criteria; purple cells indicate non-significant windows. Download Figure 5-2, TIF file. [file eneuro-13-ENEURO.0254-25.2026-s009.tif]

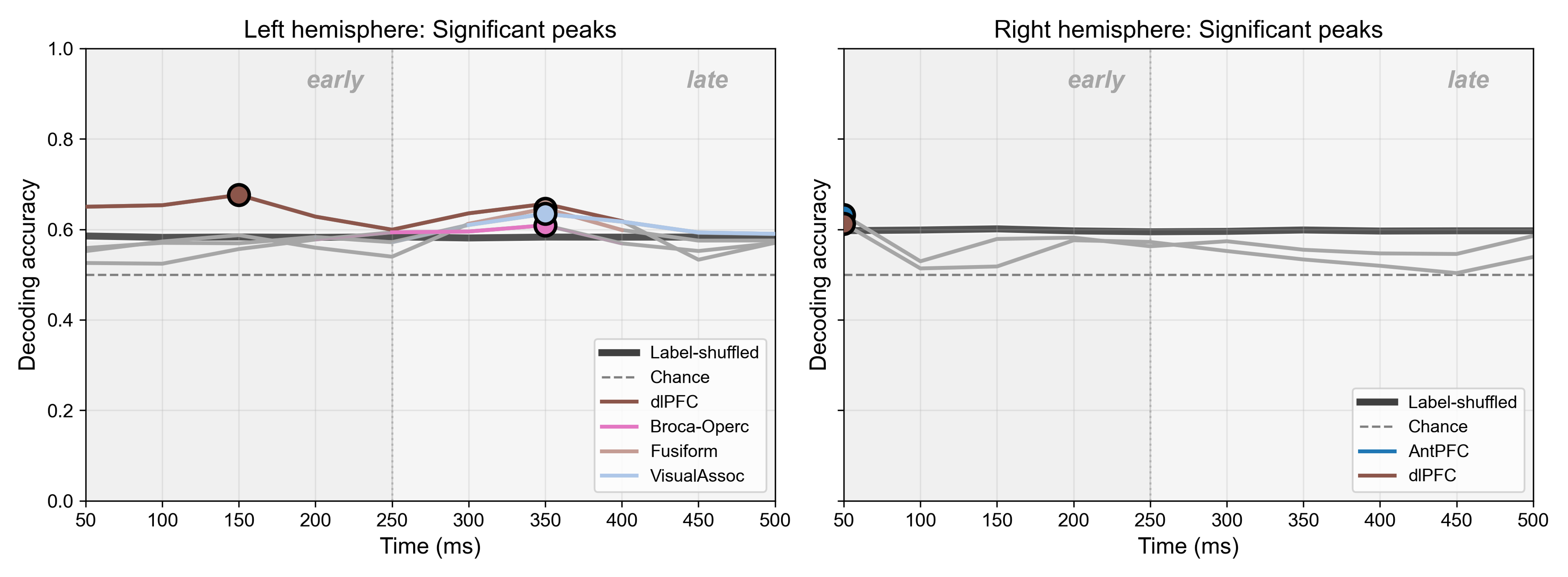

Supplement: Figure 5-3 — Decoding accuracy after excluding number words. Time-resolved decoding results for the left and right hemispheres after removing numerals from the non-body-part category. The number of body-related trials was randomly resampled (500 iterations) to match the reduced control set. Colored lines show decoding accuracy (± SEM) for significant Brodmann areas; the dark gray line indicates the mean shuffled control (95th percentile ± SEM). The overall spatiotemporal pattern remained consistent with the full analysis, showing early bilateral dorsolateral prefrontal and later left fusiform and pars opercularis activity. Because excluding number words reduced the number of available trials by nearly half, fewer Brodmann areas reached statistical significance, reflecting lower statistical power rather than a change in the underlying decoding pattern. Download Figure 5-3, TIF file. [file eneuro-13-ENEURO.0254-25.2026-s010.tif]

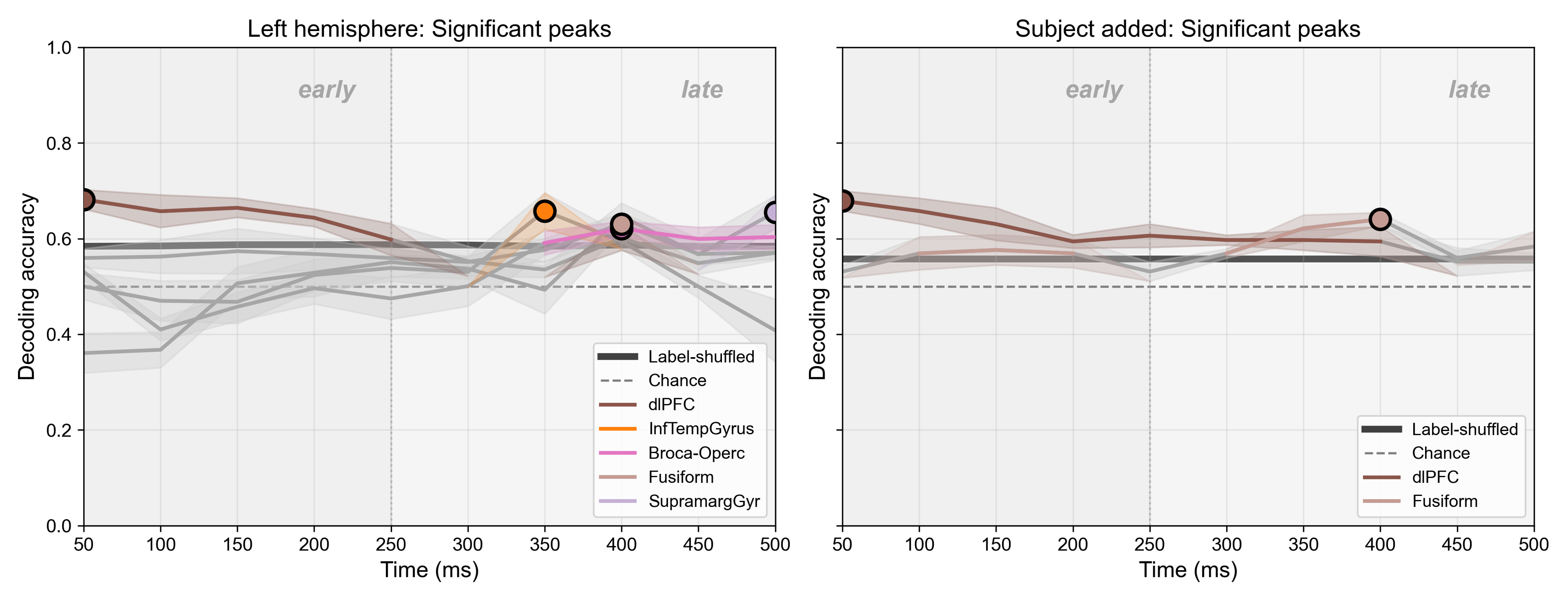

Supplement: Figure 5-4 — Replication of left-hemisphere decoding peaks after inclusion of an additional subject. Time-resolved decoding results in the left hemisphere are shown before (left panel) and after (right panel) inclusion of an additional subject, using identical preprocessing, decoding, and permutation-based statistical procedures. Circles indicate peak time points that survived the label-shuffled permutation test (FDR-corrected) and time-shuffle threshold. The temporal profile and regional pattern of significant peaks, particularly in dlPFC and fusiform cortex, remain consistent after inclusion of the additional dataset. Download Figure 5-4, TIF file. [file eneuro-13-ENEURO.0254-25.2026-s011.tif]

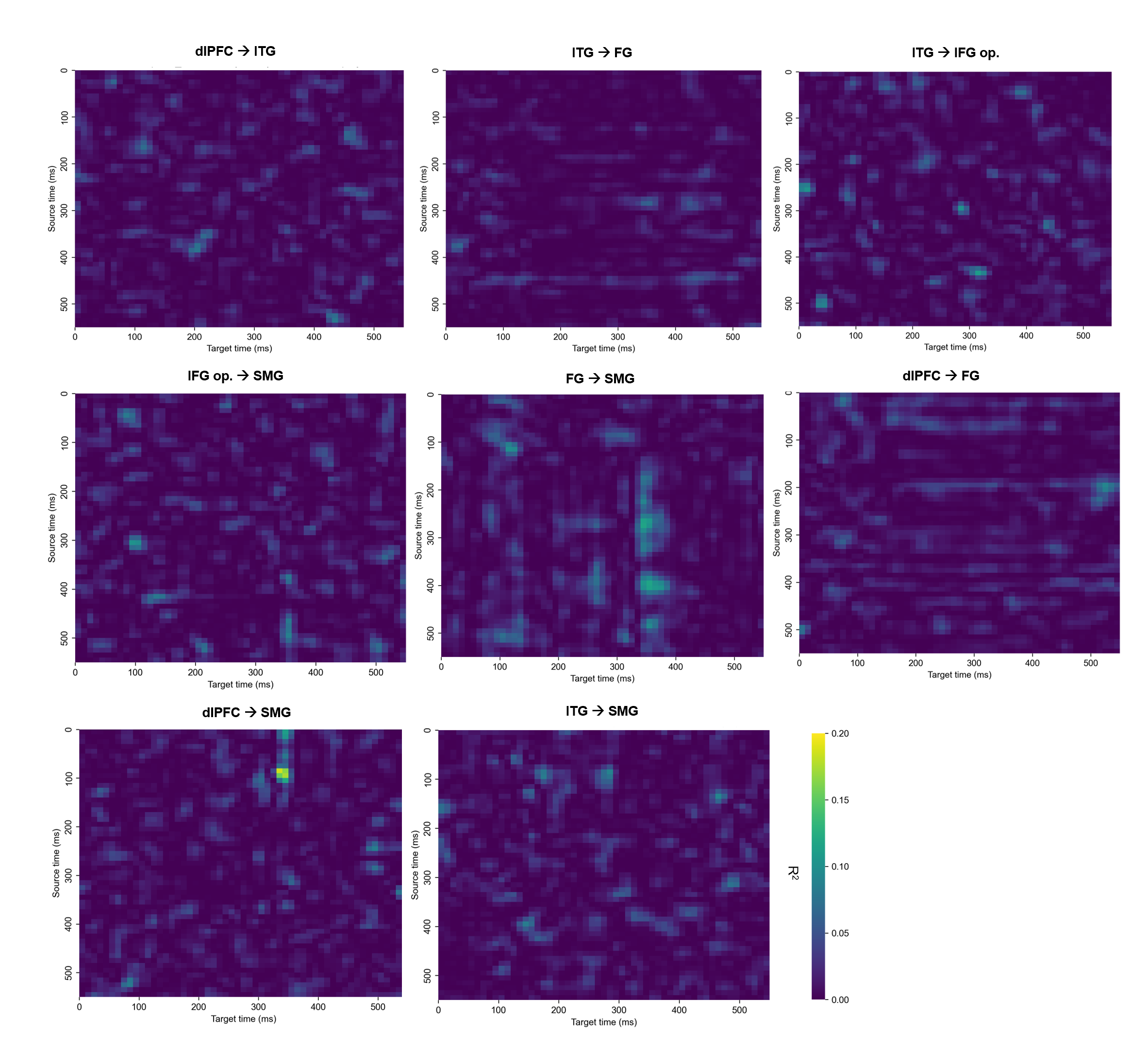

Supplement: Figure 7-1 — Cross-temporal regression results for the combined-category analysis. Heat maps display R² values across source-time × target-time pairs for left hemisphere BA pairs. Only values exceeding the 95th percentile of 1,000 permutation-based null distributions are shown. Titles indicate source → target direction. Download Figure 7-1, TIF file. [file eneuro-13-ENEURO.0254-25.2026-s012.tif]

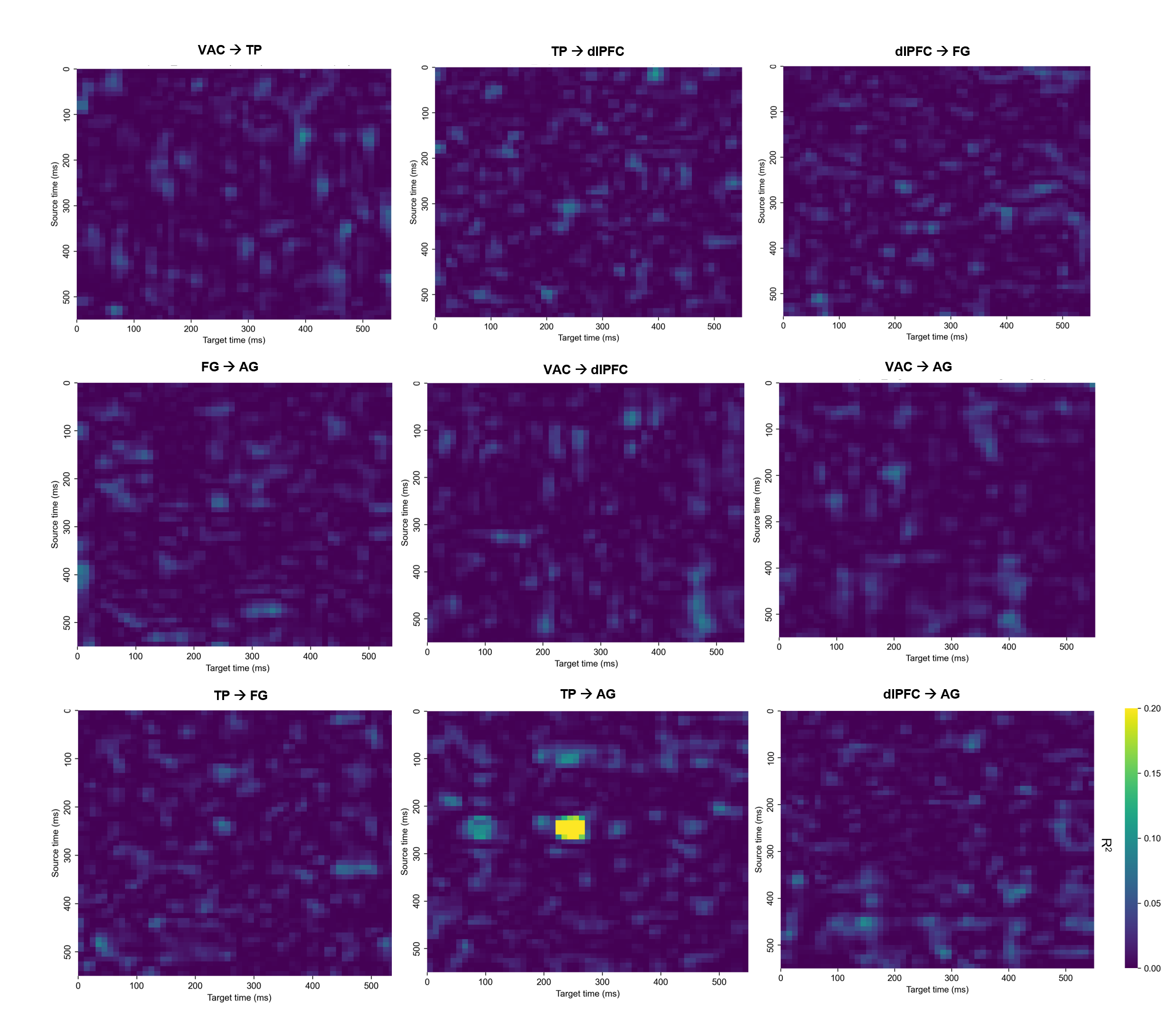

Supplement: Figure 8-1 — Cross-temporal regression results for the combined-category analysis. Heat maps display R² values across source-time × target-time pairs for right hemisphere BA pairs. Only values exceeding the 95th percentile of 1,000 permutation-based null distributions are shown. Titles indicate source → target direction. Download Figure 8-1, TIF file. [file eneuro-13-ENEURO.0254-25.2026-s013.tif]
