# Supplementary material for: Spatiotemporal Dynamics in Prespeech Semantic Category Decoding: An Intracranial EEG Study
Source: eNeuro. 2026 Apr 21;13(4):ENEURO.0254-25.2026. doi: 10.1523/ENEURO.0254-25.2026 (PMC13116012; doi:10.1523/ENEURO.0254-25.2026)
Supplement: Table 3-1 — Electrode coverage in occipitotemporal regions overlapping with the extrastriate body area (EBA), confirmed across subjects included in the decoding analyses. Download Table 3-1, DOCX file. [file eneuro-13-ENEURO.0254-25.2026-s014.docx]

**Table 3-1.**

| Subject | Hemisphere | Channels | Brodmann Area | MNI (x, y, z) range |
| --- | --- | --- | --- | --- |
| 7 | Left | 54–56, 60–63, 70–71 | Fusiform / Visual association cortex (BA 19/37) | (–60 to –50, –70 to –50, –10 to 5) |
| 12 | Right | 21–22 | Fusiform / Middle temporal gyrus (BA 37/21) | (60–70, –55 to –40, –5 to 5) |
